# Methyltransferase-like proteins in cancer biology and potential therapeutic targeting

**DOI:** 10.1186/s13045-023-01477-7

**Published:** 2023-08-02

**Authors:** Ya-Nan Qi, Zhu Liu, Lian-Lian Hong, Pei Li, Zhi-Qiang Ling

**Affiliations:** 1 Department of Pathophysiology, School of Basic Medical Sciences, Zhengzhou University, Zhengzhou, 450052 P.R. China; 2 Zhejiang Cancer Institute, Zhejiang Cancer Hospital, No.1 Banshan East Rd., Gongshu District, Hangzhou, 310022 Zhejiang P.R. China; 3 Hangzhou Institute of Medicine (HIM), Chinese Academy of Sciences, Hangzhou, 310018 Zhejiang P.R. China

**Keywords:** METTLs, Methyltransferase, Cancer biology, Therapeutics, Molecular mechanism

## Abstract

RNA modification has recently become a significant process of gene regulation, and the methyltransferase-like (METTL) family of proteins plays a critical role in RNA modification, methylating various types of RNAs, including mRNA, tRNA, microRNA, rRNA, and mitochondrial RNAs. METTL proteins consist of a unique seven-beta-strand domain, which binds to the methyl donor SAM to catalyze methyl transfer. The most typical family member METTL3/METTL14 forms a methyltransferase complex involved in *N*6-methyladenosine (m6A) modification of RNA, regulating tumor proliferation, metastasis and invasion, immunotherapy resistance, and metabolic reprogramming of tumor cells. METTL1, METTL4, METTL5, and METTL16 have also been recently identified to have some regulatory ability in tumorigenesis, and the rest of the METTL family members rely on their methyltransferase activity for methylation of different nucleotides, proteins, and small molecules, which regulate translation and affect processes such as cell differentiation and development. Herein, we summarize the literature on METTLs in the last three years to elucidate their roles in human cancers and provide a theoretical basis for their future use as potential therapeutic targets.

## Introduction

**Fig. 1:**
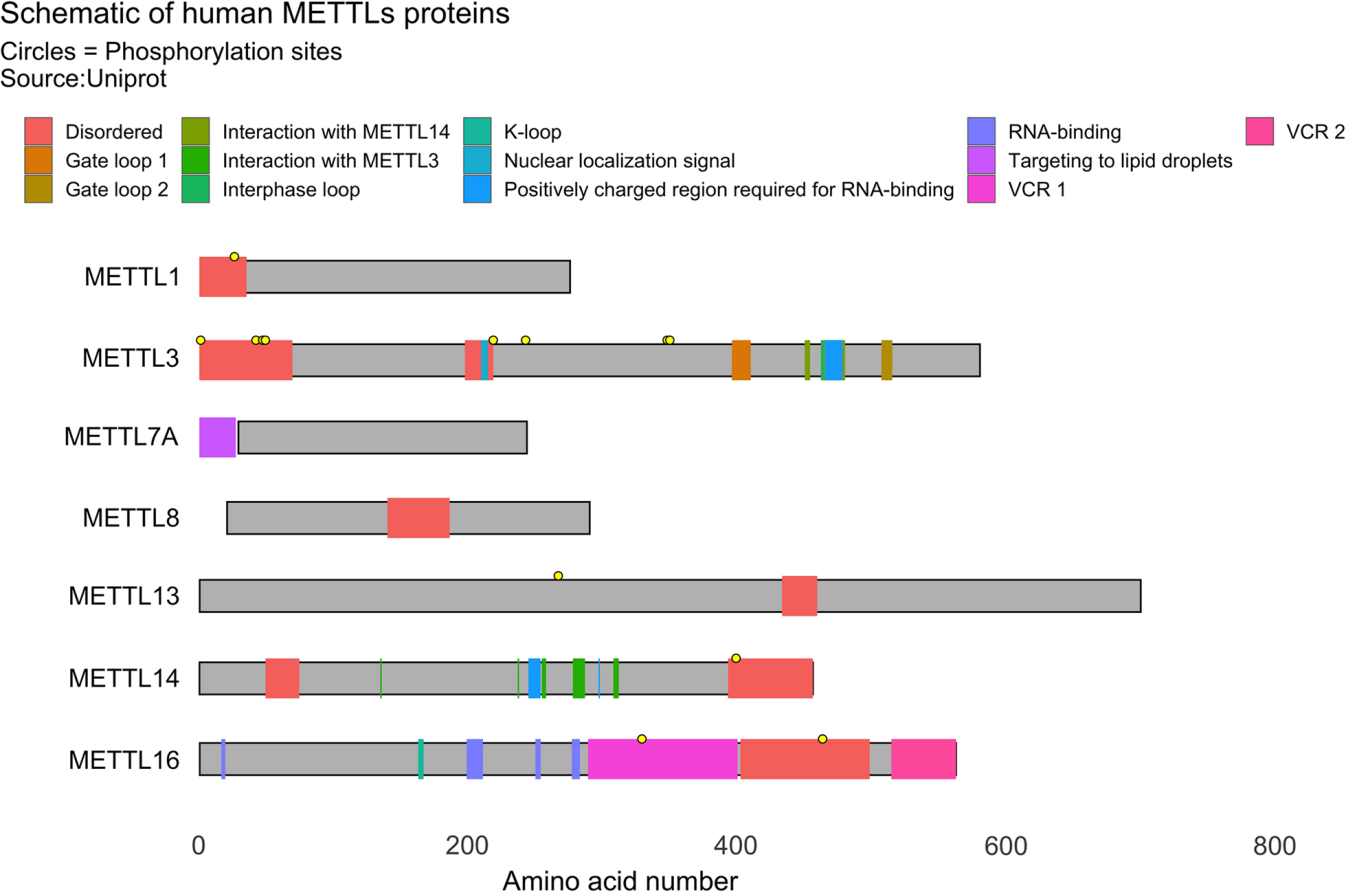
The protein structures of a subset of some METTLs family members whose protein structures are known based on data obtained from the Uniprot Protein Database

Methyltransferase-like (METTL) proteins belong to a sub-family of seven-beta-strand (7BS) methyltransferases with the function of methylating their substrates. METTLs protein and SET-domain (Su(var)3–9, enhancer-of-zeste, trithorax) protein methyltransferases are the two major family members of methyltransferases [1]. METTLs contain a conserved *S*-adenosyl methionine (SAM)-binding domain that can link to the methyl donor SAM to catalyze methyl transfer to different substrates (Fig. 1). Methylation modifies nucleic acids and proteins, regulating transcript splicing, translation, stem cell differentiation, and mitochondrial oxidative phosphorylation, influencing tumor development, tumor metabolic reprogramming, and impacting sensitivity to chemotherapy. The METTL3 and METTL14 among 34 known human METTLs [2] are more studied and involved in m6A modification. In addition to these two methyltransferases, there are six methyltransferases involved in m6A modification [3–8], and three involved in m3C modification [9, 10]. METTL15 is involved in m4C modification [11], and METTL1 is involved in m7G modification [12], Some methyltransferases mainly localized in mitochondria perform DNA and RNA methylation modifications, affecting mitochondrial metabolism.

**Fig. 2:**
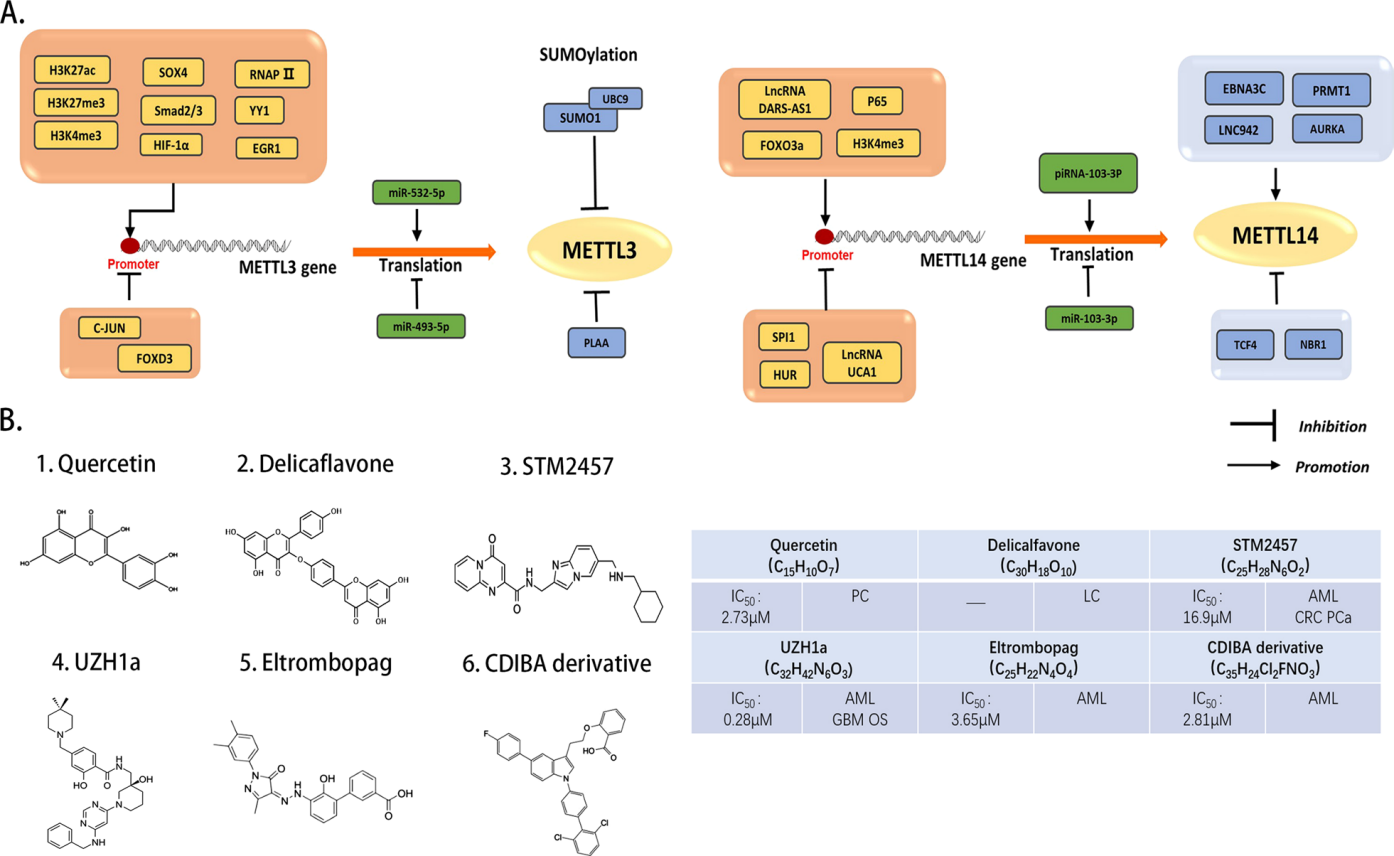
Mechanisms regulating the expression levels of METTL3 and METTL14 and small molecule inhibitors of their complexes. **A** Molecules that are known to regulate the expression level of METTL3/METTL14. Orange boxes represent regulation of METTL3/METTL14 from the transcriptional level, inhibiting or promoting their transcription; green boxes represent regulation of METTL3/METTL14 from the translational level, inhibiting or promoting their mRNA translation; blue boxes represent regulation of METTL3/METTL14 from the protein level, inhibiting or promoting their protein activity. **B** Chemical structures of current METTL3-14 inhibitors, half-maximal inhibitory concentration (IC50) and validated cancer types of METTL3/14 inhibitors. The effects of these small molecule inhibitors were derived from cellular assays, except for quercetin and eltrombopag for which no clinical trials have been reported. PC: pancreatic cancer, LC: lung cancer, PCa: Prostate cancer, CRC: Colorectal cancer, GBM: glioblastoma, OS: Osteosarcoma, AML: Acute myelocytic leukemia

METTL family proteins have a wide range of substrates, including ribosomal RNA, mtDNA, mRNA, and lncRNA, and are involved in several biological processes. They can play either pro- or anticancer roles in cancer (Table 1). Most of the METTL family members controlling tumor development have been studied, some methyltransferases have been used as predictors of prognosis and overall survival of tumor patients, and several small molecular inhibitors have been developed against METTL3 function and found effective in ex vivo experiments (Fig. 2B). However, the role of some family members in tumor pathogenesis is still unclear, except for associations with stem cell development. Their regulatory role in tumors needs to be further explored in the future. This review describes the literature evidence demonstrating the role of METTL family proteins in tumor development, and these pieces of evidence aid in developing targeted cancer therapeutics in the future.

**Table 1:** METTL family members with known roles in cancer

METTL family member	Enzymatic function	Role	Substrates
METTL1	*N*7-methylguanine	Oncogene/tumor suppressor	miRNA, tRNA, mRNA
METTL2A/B	*N*3-methylcytidine	Oncogene	tRNA
METTL3/14	*N*6-methyladenosine	Oncogene/tumor suppressor	mRNA, tRNA, pri-mRNA, lncRNA, snRNA, rRNA, miRNA
METTL4	*N*6-methyladenosine	Oncogene/tumor suppressor	mtDNA, miRNA, snRNA
METTL5	*N*6-methyladenosine	Oncogene	18S rRNA
METTL6	*N*3-methylcytidine	Oncogene	tRNA
METTL7A/B	*N*6-methyladenosine	Oncogene	LncRNA, mRNA
METTL8	*N*3-methylcytidine	Oncogene	mt-tRNA, mRNA
METTL9	Histidine methylation	Oncogene	HxH motif substrates
METTL11A	*N*6-methyladenosine	Oncogene	RCC1, RB
METTL12	Lysine methylation	Unknown	
METTL13	eEF1A methylation	Unknown	
METTL15	*N*4-methylcytidine	Unknown	12S RNA
METTL16	*N*6-methyladenosine	Oncogene	mRNA, lncRNA, snRNA U6
METTL17	*N*5-methylcytosine *N*4-methylcytidine	Unknown	mt-rRNA
METTL18	Histidine methylation	Unknown	
METTL20	Lysine methylation	Unknown	
METTL21A/C/D	Lysine methylation	Unknown	
METTL21B	Lysine methylation	Tumor suppressor

## Genomic information and basic function of METTLs

### METTL1

Methyltransferase-like 1 (METTL1) is located in the (12q13-14) region of chromosome 12 [13] in the human genome. Based on the results of HPA database, the expression level of METTL1 protein is the highest in normal human pancreas, kidney, urinary bladder and epididymis tissues, the lowest in brain tissues, and almost no expression in eye tissues. The METTL1 primarily catalyzes *N*7-methylguanosine (m7G) methylation by forming a complex with WD repeat domain 4 (WDR4) commonly in tRNA, miRNA, mRNA, and rRNA. In mRNA, METTL1/WDR4 adds an m7G onto mRNA, affecting the translation efficiency of mRNA, and in tRNA, m7G prepares the tRNA for synthesizing Arg-TCT-4-1 [12]. The tRNAs act as adaptor molecules, promoting the translation of Arg TCT-4-1, and these tRNAs with m7G modification mainly target mRNAs enriched in AGA [14]. METTL1 also mediates m7G methylation of miRNAs, enhancing the maturation of the pri-miRNA by disrupting its repressive secondary structure. The METTL1 augments let-7 miRNA family processing by disrupting the repressive secondary structure, and the METTL1-mediated m7G modification serves as a novel RNA modification pathway in regulating biogenesis and cell migration [15]. Additionally, tRNA with m7G modification stabilizes the mRNA against decay by prolonging the role of ribosomes in mRNA translation. The modified tRNA selectively regulates the oncogenic gene mRNA translation efficiency and promotes the mRNA translation of cell cycle-related regulators, thereby increasing the expression levels of these proteins through the mRNA "translatome" and remodeling of the mRNA "translatome" drives oncogenic transformation, thus inducing cancer development [15, 16].

### METTL3

**Fig. 3:**
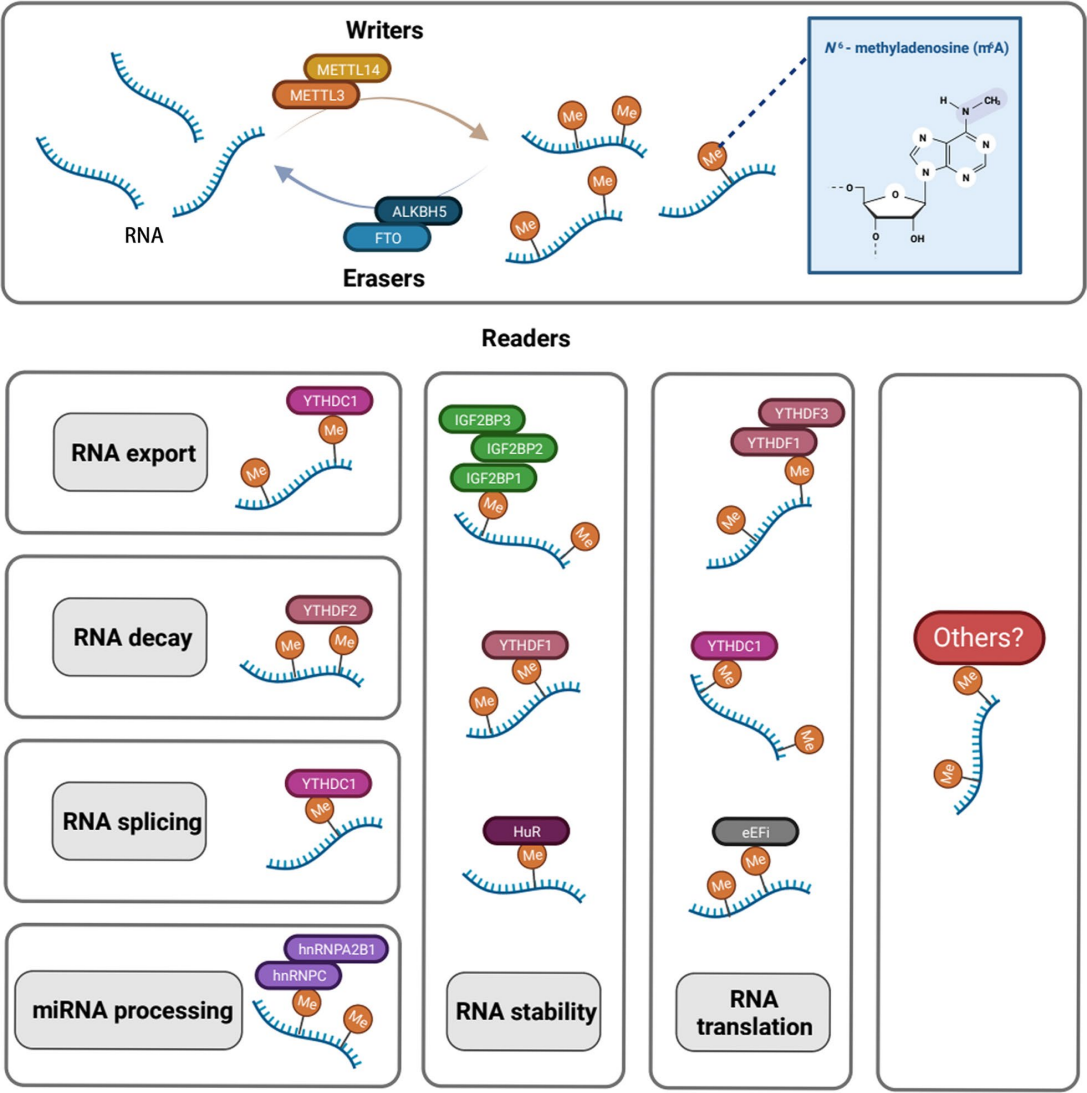
The mechanism of *N*6-methyladenosine (m6A) methylation. Introduction of the main members involved in m6A modifications. METT3 and METTL14 mainly catalyze the formation of m6A RNA modification; FTO and ALKBH5 are the major demethylating molecules; The m6A-modified RNA reader proteins include IGF2BP1/2/3, YTHDF1/2/3, YTHDC1/2/3, HuR, hnRNPA2BA, hnRNPC, eEFi, etc. M6A modification modulates RNA export, RNA decay, RNA splicing, miRNA processing, RNA stability, RNA translation

Methyltransferase-like 3 (METTL3) is the most studied member of the METTL protein family located on the human chromosome 14q11.2. METTL3 is expressed in most normal tissues, with high expression in lymphoid tissues, testis, parathyroid gland, prostate and fallopian tube tissues, and the expression of METTL3 is low in esophagus tissues, stomach tissues and gall bladder. Its full-length protein has 580 amino acids, and its major functional regions include two adjacent target-recognition zinc finger structures and a methyltransferase region [16]. The METTL3 binds to the methyl donor *S*-adenosylmethionine (SAM) and catalyzes the methyl transfer with METTL14, WTAP, ZC3H13, and other molecules, forming m6A methyltransferase complex (MTC) [16]. In 1997, researchers first discovered that the m6A methyltransferase in Hela cells consists of two protein components, MT-A and MT-B, and that the 70KDa subunit of MT-A has a SAM-binding region, thus confirming that MT-A, encoded by the METTL3 gene, is the key catalytic subunit in MTC [17]. METTL14 activates the construction of the RNA-binding scaffold and promotes the binding ability of the complex to the target RNA [17, 18]. METTL3 is responsible for 95% of m6A modifications of mRNA [19], and it performs m6A modifications on a variety of RNAs other than mRNA, including tRNA, snRNA, pre-miRNA, and lncRNA. The m6A modification regulates RNA maturation, translation, shearing, stability, and degradation, wherein METTL3 controls noncoding RNAs by interacting with microprocessor protein DGCR8 to promote pri-miRNA maturation [20] (Fig. 3). DNA methylation may bolster carcinogenesis. The m6A not only acts as an RNA regulatory modifier but also participates in regulating DNA demethylation. The m6A modifications mediated by METTL3 and acted upon by the m6A "reader" FMR1 autosomal homolog 1 (FCR1) and DNA 5-methylcytosine dioxygenase TET1, cause DNA 5-methylcytosine (5mC) demethylation, chromatin accessibility, and gene transcription [21]. The METTL3 also controls the telomere stability of cancer cell chromosomes by its m6A modification mechanism. Telomeric repeat-containing RNA (TERRA) is a long-stranded noncoding RNA transcribed from telomeres, which promotes homologous recombination by forming an R-loop and activating alternative lengthening of telomeres (ALT) pathway to maintain telomere and chromosome stability in cancer cells, resulting in uncontrolled proliferation of tumor cells. The m6A modification on TERRA is catalyzed by METTL3, recognized by increased expression of YTHDC1 [22].

In addition to the catalytic activity of METTL3 on the m6A modification, Lin and Choe et al. found that cytoplasmic METTL3 enhanced the expression of human lung cancer oncogenes, such as EGFR and TAZ [23]. This oncogene activation may be due to the interaction of the cytoplasmic METTL3 with translation initiation factors, which are essential for the formation of eukaryotic translation initiation complexes [24]. Additionally, the METTL3 interacts with poly(A)-binding protein cytoplasmic 1 (PABPC1) to stabilize the RNA loop structure by promoting PABPC1 and eukaryotic translation initiation factor 4E (eIF4F) binding, thereby preferentially promoting RNA translation of epigenetic factors without m6A modification. This regulatory function enhances tumor progression by mechanisms independent of m6A methylation [24].

Although METTL3 plays critical roles in the development of several diseases, METTL3 exerts some essential functions to ensure animal survival, such as regulation of skeletal muscle differentiation [25] and activation of mammalian spermatogenesis. METTL3 deficiency results in m6A methylation defect and spermatogonial stem cell (SCC)/progenitor cell homeostasis disruption [26].

### METTL4

METTL4 is homologous to the MT-70A subunit involved in m6A modification and mediates *N*6-adenosine methylation of RNA and DNA in various eukaryotic organisms [4, 27]. *N*6-methylation of 2ʹ-O-methyladenosine (m6Am) is present not only in the first transcribed nucleotide of mRNAs but also inside the U2 snRNAs. The identification of the m6Am-modified methyltransferase can explore its function in RNA processing. METTL4, a methyltransferase that directly adds Am in the U2 snRNA, is localized in the nucleus, and its conserved catalytic domain is critical for the m6Am modification by U2 snRNA. In the absence of METTL4, the lacking of m6Am on U2 snRNA alters the splicing of pre-mRNA transcripts [28]. The expression of METTL4 is particularly high in testis, breast, and urinary bladder tissues, with moderate levels present in lung, esophagus tissues, stomach, colon, and liver, while it is low in prostate tissues and almost absent in ovary tissues.

METTL4 is also involved in m6A modification and regulates iron death in hepatic stellate cells (HSC). The m6A modification was significantly increased after treatment of HSC with sorafenib, erastin, and RSL3. With these treatments, the demethylase FTO was downregulated and the expression of METTL4 was significantly upregulated, but not METTL3 or METTL14. The mechanism behind this consequence may be due to m6A modification of BECN mRNA by METTL4 and FTO, activating the autophagic pathway and enhancing iron death [29]. The m6A modification of microRNA by METTL4 affecting the expression of intermediate molecules during the processing and maturation of several vasoactive-related miRNAs [30], which showing significance for elucidating the mechanisms of angiogenesis and cancer development by angiogenesis.

METTL4 localized in the nucleus is involved in m6Am methylation on U2 snRNA, and METTL4 aggregated in the mitochondria mediates the 6 mA methylation of mtDNA. The 6 mA methylation in the promoter region of mtDNA represses the mitochondrial DNA transcription by preventing the binding to the mitochondrial transcription factor A (TFAM). The higher levels of 6 mA in mitochondrial DNA under hypoxic conditions impact the mitochondrial stress response [31]. Aging, metabolic diseases, and cancers alter the expression of mtDNA, and cancer cells obtain energy through mitochondria to support their uncontrolled growth. Starting from the 6 mA methylation of mtDNA, regulating the transcription of mitochondrial DNA, and shutting off the energy supply to the cancer cells may be used in the development of new cancer therapies. Inhibitors that can specifically target mtDNA have been designed, and they have been shown effective in slowing down tumor growth without affecting healthy cells in mice models [32]. Additionally, METTL4 catalyzes nuclear *N*6-deoxyadenosine methylation in a hypoxic environment and induces tumor metastasis by activating metastasis-related genes [33], showing the potential value of METTL4 as a hypoxia-related tumor therapeutic target to inhibit tumor metastasis.

### METTL5

METTL5 exerts specific activity on the UAACA motif of 18S A1832 [3, 34], modifying the conformation between ribosomal RNA and mRNA, and regulating ribosomal translation through m6A modification in this region. The expression level of METTL5 is highest in the tissues of stomach, liver, kidney, pancreas, and gallbladder. In Caenorhabditis elegans (C. elegans), lack of METTL5 homologs impairs the translation, and in several breast cancer cell lines, METTL5 deletion causes apoptosis as well as cell cycle arrest [3]. However, m6A modification of 18S RNA by METTL5 impacts the differentiation of mouse embryonic stem cells (mESCs). The m6A modification of 18S RNA by METTL5 triggers the efficient translation of the F-box and WD repeat domain (FBXW7), and METTL5 deletion reduces the level of FBXW7, accumulating its substrate c-MYC. The overall translation rate is reduced in mESCs, the differentiation potential of mESCs is impaired, and mice exhibits many morphological and behavioral abnormalities [34, 35]. Some tumor cells are due to abnormal differentiation of normal cells, so the role of METTL5 in embryonic stem cell differentiation provides a theoretical basis for exploring its role in tumorigenesis. Mutations in human METTL5 cause intellectual disability, microcephaly, and facial dysmorphism, whereas the knockdown of METTL5 in mESCs provokes craniofacial and neurodevelopmental abnormalities, showing a relationship between dysregulation of m6A modification of 18 s RNA and neurodevelopment and function [36]. This consequence reveals an important function of METTL5 in catalyzing 18S RNA m6A modification and regulating normal cell differentiation.

Previous studies demonstrated that METTL5 is an oncogene in pancreatic cancer that promotes cell proliferation, migration, invasion, and tumorigenesis. The oncogene METTL5 may increase the translation of c-MYC, and METTL5 and its cofactor TRMT112 may promote pancreatic cancer progression by regulating its translation through m6A modification of the 5'UTR and coding DNA sequence region (near the 5'UTR) of c-MYC [37, 38].

### METTL2A/B and METTL6

METTL2A/B, METTL6, and METTL8 are involved in m3C modification on eukaryotic tRNAs to maintain anticodon base repair and folding functions [9, 10], and provide a regulatory mechanism for gene expression, translation, cellular homeostasis, and tumor growth [39]. METTL2A is involved in m3C modification in breast cancer, has a high frequency of amplification (7%), and is an independent predictor of poor overall survival. METTL2A activates cell proliferation and cellular DNA synthesis pathways in BRCA cells [40]. RNA processing converts primary transcript RNA into mature RNA and altered RNA drives tumorigenesis and progression or generates a therapeutic sensitive opportunity. The expression levels of 10 RNA processing factors (including TSEN54, POLDIP3, SUGP1, RBMS1, ZBTB7A, METTL2B, CACTIN, TGFB1, PWP2, and TRUB2) in gastric cancer were correlated to the prognostic characteristics. The patients with low-risk scores had better clinical outcomes [41]. METTL6 is a key regulator of tumor cell growth [39], which is significantly upregulated in HCC tissues, promoting cell proliferation, migration, and invasion [42]. METTL6 affects tumor in vitro and cell growth in mouse PDX models, and deletion of METTL6 affects cellular translation [43].

METTL2A is generally expressed at low levels in most tissues, whereas METTL2B exhibits higher expression in liver and skeletal muscle tissue. The expression level of METTL6 is low tissue specificity, with the highest expression observed in testis and lower expression in gastrointestinal tract.

### METTL8

METTL8 is a mitochondrial protein involved in m3C modification on eukaryotic tRNAs and promotes the position C32 of the mt-tRNA^Ser (UCN)^ and 3-methyl-cytidine (m3C) methylation of mt- tRNA^Thr^. The expression level of METTL8 is highest in esophagus tissues, followed by urinary bladder, and lowest in pancreas tissues. Cellular respiration is decreased in METTL8 knockdown cells, and in pancreatic cancer, higher levels of METTL8 are associated with lower patient survival and enhanced respiratory chain activity [44, 45]. Considering the complexity of mitochondrial metabolism, METTL8 should be used as an anticancer target with caution [46]. Additionally, SUMOylation directs METTL8 to the nucleolus and regulates the localization of R-loops in the nucleus, especially the nucleolus, through its m3C catalytic activity [47], and the mis-regulation of R-loops is associated with DNA damage, excessive recombination, genomic instability, and defective transcriptional elongation [48]. STAT3 positively controls METTL8 expression in mice ESCs, and subsequently, METTL8 regulates the mouse ESC differentiation through the JNK signaling pathway [49].

### METTL14

METTL14 lacks catalytic activity due to the absence of *S*-adenosylmethionine (SAM)-binding sites [50, 51]. However, it forms a heterodimer with METTL3, which has methyltransferase activity. The METTL14 is responsible for substrate recognition and methyl group localization in the heterodimer by serving as a scaffold for RNA binding and dimer structure maintenance [52–54]. This RNA-binding capability stabilizes RNA G-quadruplex (rG4) structures, which are non-canonical four-stranded structures generated by G-rich sequences, through the METTL14 RGG repeats [55]. Until now, many elucidations regarding the relationship between METTL3-METTL14 complex and malignancies have focused on the RNA m6A modification, and its function independent of m6A have remained poorly understood [56]. A hypothesis generated was that the METTL3-METTL14 complex promoted a senescence-associated secretory phenotype (SASP) without involving m6A modification to produce a pro-tumor effect [56]. METTL14 is widely expressed in various tissues, with the lung tissues and testis tissues showing the highest abundance of METTL14 protein and transcript expression. The gastrointestinal tract, pancreas, kidney, urinary bladder, female tissues and liver tissues also exhibited significant mRNA expression; however, the protein expression of METTL14 is low in liver tissues, and the expression of METTL14 is lowest in brain tissues.

### METTL16

Methyltransferase-like protein 16 (METTL16) located in the 17p13.3 chromosome is an RNA methyltransferase containing 562 amino acids. METTL16 is abundantly expressed in tissues of female genital, lymphoid, and parathyroid gland, while brain and gastrointestinal tract tissues show lower levels of expression. It consists of an N-terminal methyltransferase structural domain (MTD) and a C-terminal structural domain having two vertebrate-conserved regions (VCRs) [57]. METTL16 mainly mediates m6A modification and differs from METTL3/METTL14 in performing m6A modification in U6 small nuclear RNA (U6 snRNA) as well as SAM synthetase pre-mRNA. METTL16 also controls the m6A epi-transcriptome by SAM homeostasis, and its functional N-terminal MTD region interacts with substrates as well as coenzymes; its conserved VCRs are intercalated with U6 snRNA, catalyzing the m6A modification in position 43 (m6A43) of U6 snRNA [58, 59]. METTL16 in the nucleus serves as an m6A "writer" for m6A modification of target RNAs, whereas METTL16 in the cytoplasm binds directly to the eukaryotic initiation factors 3a and 3b as well as ribosomal RNA through its methyltransferase domain to assemble a translation complex in an m6A-independent manner [7].

Furthermore, METTL16 is an essential mammalian protein, and its knockdown in cell lines and mice leads to embryonic lethality [60]. During erythroid differentiation of myeloid cells, genetic integrity is critical in maintaining normal differentiation, and METTL16 regulates the differentiation. Along with the MTR4-nuclear RNA exosome complex, the DNA m6A modification promotes the expression of repair-related transcripts (e.g., Brca2 and Fancm) and coordinates DNA repair in erythroid progenitors to protect genomic integrity. METTL16-deficient erythroid cells show defective differentiation and DNA damage and activate apoptosis [61]; therefore, alterations in METTL16 expression or its function may be associated with malignant hematologic tumors, laying a theoretical foundation for future studies on the potential therapeutic value of METTL16 in relevant cancers.

### Others

**METTL9** is an N1 histidine methyltransferase, recognizes the His-x-His motif of the substrate, mainly involved in 1MH (1-methylhistidine) modification, and the deletion of METTL9 inhibits tumor growth and effectively induces anti-tumor immunity [62, 63]. METTL9 has low tissue specificity, with the highest expression observed in kidney and the lowest in liver and testis. **METTL12** is a mitochondrial methyltransferase that modifies the methylation of the lysine-368 site of citrate synthase in mitochondria to regulate its activity [64, 65]. The expression of METTL12 is low or absent in many normal organ tissues. **METTL15** is the major *N*4-methylcytidine (m4C) methyltransferase in humans and methylates the C839 site in the mitochondrial 12S RNA. Lack of METTL15 reduced de novo synthesis of mitochondrial proteins which is important for maintaining the stability of the translation process in mitochondria [11, 66]. **METTL17**, a regulator of mitochondrial ribosomal RNA modification, is localized in mitochondria through its N-terminal sequence and is required for the translation of mitochondria-encoded genes and differentiation of mESCs. METTL17 deficiency leads to defective ribosomes in mitochondria and impairs translation of mitochondrial proteins, affecting oxidative phosphorylation, cellular metabolism, and normal differentiation of mESCs [67]. The expression level of METTL17 in normal tissues is low, and the content of METTL17 in cerebellum is relatively high. **METTL18** is also a histidine-specific methyltransferase that targets 60S ribosomal protein L3 (RPL3) and affects ribosome biogenesis and function [68–70]. The expression of METTL18 is abundant in parathyroid gland tissues. **METTL20** is a mitochondrial lysine methyltransferase that targets the *β* Subunit electron transfer flavoprotein (ETF*β*) and regulates its activity [71–73]. The expression of METTL20 is lowly expressed in normal tissues.

## Expression of METTLs in human cancers and outcome

### METTL1

METTL1 is frequently overexpressed in a variety of cancers, such as hepatocellular carcinoma (HCC) [74, 75], bladder cancer (BC) [76], head and neck squamous cell carcinoma (HNSCC) [77], esophageal squamous cell carcinoma (ESCC) [78, 79], nasopharyngeal carcinoma (NPC) [80], and gastric cancer (GC) [81], and its high expression is often associated with poor prognosis of patients. A recent study showed that high METTL1 expression is linked to abnormal proliferation of tumor cells, tumor invasion, metastasis and progression, chemotherapy resistance, immunosuppression, and dysregulation of autophagy. Tumor cell lines exhibiting higher METTL1 expression are more sensitive to drugs targeting WNT, ERK/MAPK pathways, and chromosomal histone methylation. The patients expressing METTL1 expression are more effective with PD-L1 therapy than the less-expressing group [82].

In human induced pluripotent stem cells (hiPSCs), METTL1-mediated alterations in m7G increase the translation efficiency of marker genes, METTL1 knockdown reduces the pluripotency and slows the cell cycle in hiPSCs, in addition to accelerating the differentiation of hiPSCs to embryoid bodies (EBs) and promoting mesoderm-related genes expression [83]. This result contributes to understanding the role of METTL1 in regulating differentiation and cell stemness and provides new insights into treatment of tumors associated with vascular development.

### METTL3

Previous clinical studies in gastric cancer (GC) [84], colorectal cancer (CRC) [85], liver cancer [86–88], multiple myeloma (MM) [89], osteosarcoma (OS) [90–92], cervical cancer (CC) [93, 94], prostate cancer (PCa) [95], renal clear cell carcinoma (RCC) [96], esophageal squamous carcinoma (ESCC) [97], pancreatic cancer (PC) [24], thymic epithelial tumors (TETs) [98], acute myeloid leukemia (AML) [99], gallbladder cancer (GBC) [100], non-small cell lung cancers (NSCC) [101–103] find elevated METTL3 expression, and high expression level of METTL3 is significantly associated with more severe pathological features such as poor prognosis, clinical stage cancer, and distant metastasis. High expression of METTL3 promotes tumor cell proliferation, invasion and metastasis, and resists to chemotherapy, generating immune resistance.

However, in different classifications of the same tumor, the expression of METTL3 is different in paracancerous tissues and tumor tissues. For example, thyroid cancer (TC) is the most common endocrine malignancy and is classified into papillary thyroid cancer (PTC), follicular thyroid cancer (FTC), and anaplastic thyroid cancer (ATC) based on its degree of differentiation. The PTC and FTC account for 95% of TC and are sensitive to surgery and radiotherapy with good prognosis, however, ATC is rare and high invasiveness. In ATC, the expression of METTL3 in tumor tissues is lower than paracancerous tissues, the m6A modification by METTL3 specifically regulates the expression of DNA damage-inducible 1 homolog 2 (DDI2) gene for the proliferation of ATC cells and the METTL3 acts as a tumor promoter gene [104], but in PTC, the expression of METTL3 in cancer tissues is lower than corresponding paracancerous tissues [105, 106], and the METTL3 acts as a suppressor of PTC progression. In addition, METTL3 protein and m6A expression levels are all lower in endometrial cancer tissues [107], and METTL3 inhibits the proliferating and migrating ability of cancer cells in endometrial cancer [107].

METTL3 expression and its function are vague in the most malignant glioma, a heterogeneous group of primary CNS tumors. However, METTL3 expression is low in glioma and promotes glioma cell proliferation [108]. Further, downregulating METTL3 or METTL14 significantly promotes glioblastoma stem cell (GSC) growth, self-renewal, and tumorigenesis [109]. In contrast to this outcome, another study showed that upregulation of METTL3 [110] is positively correlated with high malignancy and poor prognosis in IDH wild-type gliomas, but not in IDH mutant gliomas [111]. METTL3 is upregulated in glioma microvascular endothelial cells (GECs), inhibiting blood tumor barrier (BTB) permeability, impeding chemotherapeutic drug penetration, and reducing the efficacy of chemotherapy [112]. Therefore, the ambiguity of METTL3 expression levels in gliomas may vary depending on the malignancy and heterogeneity of gliomas, and differences in METTL3 expression may be seen in gliomas of different stages. A more detailed delineation will aid in understanding the function of METTL3 in gliomas.

### METTL11A and METTL13

METTL11A is significantly overexpressed in cervical cancer and m6A modification of ELK3 mRNA promotes cervical cancer cell metastasis [8]. METTL13 is involved in eEF1A (eukaryotic elongation factor 1A) lysine 55 (eEF1AK55me2) modification, increasing GTPase activity, protein translation, synthesis of proteins associated with oncogenic growth signals in the presence of KRAS [113, 114] and promoting tumorigenesis in vivo. METTL13 and eEF1AK55me2 levels are upregulated in cancers and negatively associated with survival in pancreatic and lung cancer patients [115]. Hematological and neurological expressed 1 like (HN1L) gene, an oncogene in HCC, upregulated METTL13 expression in an AP-2*γ*-dependent manner and promotes the HCC growth and metastasis through the AP-2*γ*/METTL13/TCF3-ZEB1 axis [116]. In HNSCC, METTL13 mediates the Snail upregulation, inducing EMT and promoting the malignant phenotype of HNSCC [117]. However, in renal clear cell carcinoma, METTL13 can negatively regulate PI3K/AKT/mTOR/HIF-1*α* pathway and inhibits c-Myc protein expression, suppressing cell proliferation and metastasis in ccRCC [118].

### METTL14

Being a closely related gene to METTL3, the association between METTL14 and tumor progression is still controversial. Some pieces of evidence suggest that METTL14 can act as an oncogene in promoting tumor development [56, 119, 120] and leading to a poor prognosis [121, 122], but other studies show that METTL14 can function as a cancer-suppressive gene to some extent [123–125].

### METTL16

The m6A modification of cyclin D1 is increased by METTL16 to promote its mRNA stability and gastric cancer proliferation and growth. The downregulation of METTL16 inhibits the proliferation through G1/S blockade [126]. Pancreatic ductal adenocarcinoma (PDAC) is one of the most lethal cancers, and PDAC exhibiting high METTL16 expression is very sensitive to PARP inhibition treatment, especially when combined with gemcitabine. METTL16 does not depend on its methyltransferase activity, it inhibits the exonuclease activity of double-strand break repair nuclease (MRE11), thereby impeding DNA homologous recombination (HR) repair of DNA end resections, and the METTL16-RNA complex is also a potent inhibitor of MRE11-mediated DNA end resection [127, 128]. In hepatocellular carcinoma, METTL16 hinders the stability of LncRNA RAB11B-AS1 transcripts by direct binding to LncRNA BRB11B-AS1, mediating its m6A modification, resulting in tumor cell proliferation, invasion, and migration, and inhibiting tumor cell apoptosis in HCC [129].

However, METTL16 expression is positively correlated to overall survival in thyroid cancer and adrenocortical carcinoma [130]. Low expression predicts poor OS and DFS in HCC and activation of multiple metabolic pathways is associated with low METTL16 expression in HCC. Metabolic reprogramming is a hallmark alteration in cancers and METTL16 may play a role in the metabolic reprogramming of cancers [131]. METTL16 can be a protective gene and suppress OC, HCC, and endocrine tumorigenesis [130–132]. Previous studies targeting METTL16 in tumors through the mechanism of m6A modification have divergent outcomes, probably because METTL16 is very dependent on other regulatory enzymes of m6A for m6A modification. Also, METTL16 is a cofactor, and its knockdown or overexpression does not affect much the m6A modification, altering the tumor cell phenotype. However, METTL16 relies on its structural and functional region to interact with some specific RNAs [133]. MEETTL16 specifically recognizes cancer metastasis-associated LncRNA MALAT1 triple helix located at the 3' end, and the interaction remains unknown. MALAT1 interaction may be related to tumorigenesis [134, 135]. It is critical to investigate its function on tumorigenesis.

## Mechanisms for upregulation or downregulation of METTLs in cancers

### METTL1

Among several pathways controlling tumor cell migration and invasion, AKT oncogenic signaling pathway is importantly activated in cancer pathogenesis [136]. AKT can directly phosphorylate METTL1 to inhibit its enzymatic activity [137]. The over-activated AKT in cancers may reduce the expression of tumor-suppressing miRNAs containing m7G modifications, including let-7 family miRNAs, which inhibits the progression of tumors by regulating the expression of key oncogenes such as MYC, high mobility group AT-hook 2 (HMGA2), and RAS [138]. METTL1 is upregulated in multiple cancers, however, TCGA database analysis shows that METTL1 expression is not upregulated in nasopharyngeal carcinoma. The mechanism might have been due to the downregulation of transcription factor aryl hydrocarbon receptor nuclear translocator (ARNT) in nasopharyngeal carcinoma and the repressive regulation of the METTL1 promoter region. ARNT is also negatively correlated with METTL1 expression [80].

### METTL3

METTL3 is aberrantly expressed in several cancers and exploring the mechanism can aid in identifying therapeutic targets. Epigenetic activation of transcription is a critical trigger that alters gene transcription. In recent years, H3K27 acetylation (H3K27ac) in the METTL3 promoter region, and catalyzation of the acetylation by the P300/CBP complex are abnormally enriched. METTL3 promoter H3K27ac activation led to partial upregulation of METTL3 [139]. Additionally, transcriptional activator SETD1B with H3K4 tri-methyltransferase activity induces tri-methylation of lysine 4 on histone 3 (H3K4me3) in the METTL3 transcription start site and enriched the transcription [88]. The transcription factor ETS1 also recruits P300 and WDR5 to mediate lysine 4 of the histone H3 (H3K4me3) and lysine 27 on histone 3 (H3K27ac) histone modifications in the METTL3 promoter to induce METTL3 transcriptional activation [93]. METTL3 upregulated tet methylcytosine dioxygenase 1 (TET1) via m6A-YTHDF2, and then TET1 promoted H3K27me3 and H3K4me3 enrichment in METTL3 DNA and METTL3 expression by mediating DNA methylation of METTL3 [140]. Similarly, METTL3 expression in temozolomide-resistant glioblastoma cells (GBM) is elevated by temozolomide by increasing the level of histone H2K27ac activation, recruiting RNA polymerase II(RNAPII), and inducing SOX4 binding to the METTL3 promoter [141].

In addition to epigenetic modification regulating the expression of METTL3, Homeobox A10 (HOXA10) plays an important role in embryonic development and tumor progression and is enriched in the promoter region of TGFB2, activating the TGF*β*/Smad signaling pathway. Consequently, Smad2/3 expression is increased in the nucleus, binding to the promoter region of METTL3 and resulting in the upregulation of METTL3 [142]. The HDAC1/3 induces liquid–liquid phase separation of YY1, which binds to the promoter region of METTL3 as a transcription factor and promotes its expression. The HDAC inhibitors reduce the YY1 binding to METTL3 and thereby downregulate the METTL3 expression [143]. Early growth response 1 (EGR1) expression is positively correlated to METTL3, and EGR1 binds to the transcription region of METTL3 and promotes the METTL3 expression [144]. Adenosine-to-inosine deamination (A-to-I editing) catalyzes by adenosine deaminase edits the METTL3 mRNA open reading frame (ORF) near the stop codon region by acting on ADAR1, altering the binding site to miR-532-5p and increasing METTL3 translation [145]. Long-term exposure to fine particulate matter (PM2.5) can increase the risk of cancer in humans, and PM2.5 exposure is significantly associated with increased m6A methylation in bladder cancer. PM2.5 increases the binding of the transcription factor HIF-1*α*by inducing promoter hypermethylation and increasing METTL3 expression [146].

Several molecules can reduce the expression level or inhibit the m6A methyltransferase of METTL3. C-JUN binds to the METTL3 promoter to decrease the METTL3 expression and m6A modification in bladder cancer [147]. Small ubiquitin-like modifier-1 significantly inhibits the METTL3 methyltransferase activity [148]. Phospholipase A2-activating protein (PLAA) suppresses METTL3 protein expression by mediating the ubiquitination degradation pathway [149]. Maternally expressed gene 3 (MRG3) in AML upregulates miR-493-5p expression and inhibits METTL3 expression, however, in arabinocytosine (AraC) chemoresistant AML cells, MRG3 is downregulated and the inhibition of METTL3 expression is reduced [150]. In previous studies on colorectal cancer, microbes and their metabolites also regulate the METTL3 expression, and Clostridium nucleatum hinder the METTL3 transcription by activating YAP signaling in the nucleus and inhibiting FOXD3 expression [151] (Fig. 2A).

In summary, METTL3 expression is upregulated mainly by changes in its transcriptional level. Increased expression of transcription factors, increased aggregation of transcription factors in the METTL3 promoter region, increased activity of transcriptional activation molecules and activation of histones can promote METTL3 transcription; in addition, sponge adsorption of microRNA to METTL3 mRNA affects its translation and inhibits its expression, and regulation of the ubiquitination process promotes METTL3 protein degradation. In addition to changes in METTL3 expression levels, which can affect normal physiological functions and alter tumorigenesis and progression, changes in METTL3 activity also play an important role. The ubiquitin-like protein modifier SUMO1 can inhibit METTL3 enzyme activity, and some small molecules bind to the catalytically active region of METTL3 and inhibit its function.

### METTL14

A transcription factor, Spi-1 proto-oncogene (SPI1), negatively regulates the METTL14. Direct binding of SPI1 in the promoter of METTL14 has been observed, and decreased METTL14 inhibits acute myeloid leukemia (AML) cell growth and enhances myeloid differentiation through SPI1-METTL14-MYB/MYC signaling axis [152]. The transcription factor P65 upregulates METTL14 transcription by binding to its promoter region, thereby enhancing the production of cytidine deaminase (CDA) and resulting in resistance to gemcitabine treatment. Additionally, NF-B p65 directly transactivates the METTL14 gene in mice exposed to lipopolysaccharide (LPS) [153], leading to global RNA m6A hypermethylation and promoting the transition from non-alcoholic steatohepatitis (NASH) to fibrosis, a critical step in the initiation of hepatocellular cancer [154]. Another transcription factor, HuR, directly binds to the promoter of METTL14 and adversely regulates its expression [155]. In the same study, researchers discovered that the loss of transcription factor 4 (TCF4) destabilized the METTL14 protein by increasing its ubiquitination level. FOXO3a is also identified as a transcription factor that upregulates the expression of METTL14 in bladder cancer. Isorhapontigenin (ISO) causes an epithelial-mesenchymal transition (EMT) by inhibiting vimentin through METTL14 upregulation in a FOXO3a-dependent manner. Moreover, aurora kinase A (AURKA) stabilized METTL14 by preventing its ubiquitylation and degradation in breast cancer stem-like cells, and the enhanced expression of METTL14 increases DROSHA and mRNA methylation, contributing to breast cancer stem-like cell (BCSC) characteristics [156]. The exon 10 skipping of METTL14 is inhibited in pancreatic ductal adenocarcinoma (PDAC) by SRSF5 Ser250 phosphorylation, mediated by a PDAC over-expressed gene, Cdc2-like kinases 1 (CLK1). The alternative splicing (AS) pattern of the exon 10 skipping inhibition in METTL14 increases the m6A modification and enhances pancreatic cancer cell proliferation and metastasis [157]. Noncoding RNAs (ncRNAs) also regulate the expression of METTL14. LNC942 binds to METTL14 protein directly through a specific recognition sequence (+ 176–+ 265), stabilizing the expression of CXCR4 and CYP1B1 via post-transcriptional m6A methylation modification, and resulting in the development and progression of breast cancer [119]. The miR-103-3p also binds directly to METTL14 and inhibits osteoblast activity through miR-103-3p/METTL14/m6A signaling axis [158]. Autophagy plays a role in regulating METTL14, and a neighbor of BRCA1 gene 1 (NBR1) binds to METTL14, mediating its autophagic degradation. As a tumor-suppressive protein in skin cancer, decreased expression of METTL14 results in a dysfunction of global genome repair (GGR) and promotes ultraviolet B (UVB) radiation-induced skin carcinogenesis [159]. A PIWI-interacting RNA (piRNA), piRNA-14633, has been discovered as a METTL14 regulator. METTL14 was identified as a direct target gene of piRNA-14633 by dual luciferase reporter assays, and piRNA-14633 increases the METTL14 mRNA stability. The piRNA-14633 promotes the development of cervical cancer through the METTL14/CYP1B1 signaling axis dependent on m6A modification [160]. Silencing lncRNA UCA1 can lead to reduced DNA methylation of METTL14, resulting in increased METTL14 expression, decreased cancer cell proliferation and invasion, and enhanced apoptosis. [161]. Other IncRNAs, such as the aspartyl-tRNA synthetase 1 antisense 1 (DARS-AS1), an oncogenic lncRNA influences the METTL14 expression in various cancers [162].

In Epstein–Barr virus (EBV)-associated malignancies, METTL14 is upregulated through transcriptional activation by the viral-encoded latent oncoprotein Epstein–Barr nuclear antigen 3C (EBNA3C). The direct interaction between METTL14 and the unique amino domain of EBNA3C contribute to the maintenance of METTL14 stability [163]. Moreover, DNA methylation in the METTL14 promoter region may affect its expression. The lysine-specific histone demethylase 5C (KDM5C) decreases the histone H3K4me3 in the promoter region of METTL14. The knockdown of KDM5C significantly enriches the H3K4me3 at the METTL14 promoter and increases its expression [124]. The methylation in the C-terminal region of METTL14 is also functionally related. The protein arginine methyltransferase 1 (PRMT1) interacts with the C-terminal region of METTL14, resulting in METTL14 methylation. C-terminal methylation increases its affinity for RNA substrates, enhancing its RNA methylation activity [164] (Fig. 2A).

## METTLs in tumor proliferation, invasion, and metastasis

### METTL1

In hepatocellular carcinoma, m7G tRNAs are critical for tRNA function and mRNA translation. Upregulation of EGFR and VEGFA in METTL1 overexpressed cells induce the EGF/EGFR and VEGRA/VEGFR1 signaling pathway-related molecules, thereby promoting proliferation, migration, and invasion of HCC cells [74]. METTL1-mediated m7G modification promotes the translation of DNA ligase IV, enhancing the non-homologous end joining (NHEJ) repair-mediated DNA double-strand break repair, and causing resistance to radiation therapy [165]. Further, m7G tRNAs promotes the metastasis of HCC cells after radiofrequency ablation by enhancing SLUG/SNAIL translation [166]. The m7G modification of tRNAs by METTL1 selectively alter the translation of genes involved in the cell cycle and EGFR pathway, promoting tumorigenesis and progression in intrahepatic cholangiocarcinoma (ICC) [167]. METTL1 is overexpressed in gastric cancer, regulating the cell cycle through the AKT/STAT3 pathway, and METTL1 knockdown significantly inhibits GC cell proliferation [81]. In bladder cancer, high expression of METTL1 is associated with malignant tumor phenotype. BC cell proliferation, metastasis, and invasion are enhanced by m7G modifications on certain tRNAs promoting mRNA for EGFR/EGF-containing fibulin extra-cellular matrix protein 1 (EFEMP1) axis-related regulators [76]. The METTL1/WDR4 is significantly upregulated in lung cancer and negatively correlated with cancer prognosis. METTL1 aids the mRNA translation of its downstream cell cycle-related target gene cyclin D3 by increasing m7G tRNA, which induces tumor cell invasion and proliferation [168]. The m7G tRNA of METTL1/WDR4 is upregulated in Head and neck squamous cell carcinoma (HNSCC), the m7G modification increases the levels of 16 tRNAs that promote the translation of certain mRNAs through codon complementation due to specifically elevated tRNAs promoting mRNA translation of genes related to the PI3K/AKT/mTOR signaling pathway. These effects form an immunosuppressive microenvironment and induced the development and progression of HNSCC [77]. METTL1/WDR4 upregulation in esophageal squamous cell carcinoma (ESCC) elevates the m7G tRNA expression and increases the translation of oncogene for the regulatory protein of MTOR complex 1 (RPTOR)/unc-51 like autophagy activating kinase 1 (ULK1)/autophagy pathway. High METTL1 expression upregulated RPTOR, which inhibited ULK1 dephosphorylation and autophagy, promoting ESCC tumorigenesis [78, 79]. METTL1 upregulation in nasopharyngeal carcinoma (NPC), and the knockdown of METTL1 considerably inhibits cell proliferation, colony formation, migration, and invasion [80]. The tRNA m7G mediates a novel translational regulatory mechanism that connects the translation and autophagy, suggesting that METTL1 and its downstream signaling axis may be potential targets for the treatment of ESCC (Table 2).

**Table 2:** The role of METTL1 in various cancers

Role	Cancer	MTTL1 Targets	Downstream	Prognosis*	Mechanism	Cellular function	References
Oncogene	HCC	HDGF		Poor	m7G tRNAs	Proliferation	[74]
		TGF-*β*2	PMN-MDSCs	Poor	m7G tRNAs	Immunosuppressive microenvironment	[75]
		EGFR		Poor	m7G tRNAs	lenvatinib resistance	[250]
		DNA-PKcs or DNA ligase IV	NHEJ-mediated DNA DSB repair	Poor	m7G tRNAs	Radiotherapy resistance	[165]
		SLUG/SNAIL	DNA repair	Poor	m7G tRNAs	Metastasis	[166]
	NPC		WNT/*β*-catenin	Poor	m7G tRNAs	EMT, chemoresistance	[80]
	Lung cancer	Proliferation-associated mRNA		Poor	m7G tRNAs	Growth, migration, and invasion	[168]
	ICC	EGFR, cell cycle-associated mRNA		Poor	m7G tRNAs	Proliferation	[167]
		CXCL8	PMN-MDSCs	Poor	m7G tRNAs	Immunosuppressive	[232]
	Bladder cancer	EGFR/EFEMP1		Poor	m7G tRNAs	Proliferation, migration, and invasion	[76]
	ESCC	RPOTOR/ULK1		Poor	m7G tRNAs	Proliferation, autophagy	[78]
		MTORC1	ULK1	Poor	m7G tRNAs	Autophagy	[79]
	GC	Cell cycle-associated mRNA	AKT/STAT3	Poor	m7G tRNAs	Proliferation	[81]

*Represents the correlation of a higher expression level of METTL1 with a good or poor prognosis

### METTL3

METTL3 promotes cancer cell proliferation, invasion, and metastasis in several tumors. In gastric cancer, METTL3 along with m6A methylated reading protein HuR increases the expression of zinc finger MYM-type containing 1 (ZMYM1) and inhibits the transcription of cell adhesion protein E-cadherin [169]. The m6A modification also upregulates the expression of transcriptional repressors snail family transcriptional repressor 1 (Snail) and Slug [142]. Suppressed E-cadherin impacts intercellular adhesion, induced epithelial-mesenchymal transition (EMT), and promotes metastasis in gastric cancer cells. Further, METTL3 promotes the translation of sphingosine kinase 2 (SPHK2), mediating KLF transcription factor 2 (KLF2) degradation via the ubiquitin–proteasome pathway, promoting gastric cancer proliferation and metastasis [170].

METTL3-mediated m6A modification enhances the expression of LncRNA THAP7-AS1 in the presence of IGF2BP1, and LncRNA THAP7-AS1 promotes GC cell proliferation, migration, and invasion by mediating the entry of its downstream gene CUL4B into the nucleus [171]. In addition, lncRNA recruits METTL3 to increase keratin 18 (KRT18) mRNA stability and its expression levels in GC cells, activating MAPK/ERK signaling [172]. METTL3 can also stabilize DEK proto-oncogene (DEK) mRNA expression through m6A modification to promote GC cells proliferation and migration [84]. In colorectal cancer, high expression of METTL3 increases m6A modification on CRB2 mRNA, and YTHDF2 recognition modification degraded CRB2 [173]. The m6A methylation of EphA2 and vascular endothelial growth factor A (VEGFA) is recognized by IGF2BP2 and IGF2BP3, respectively, by activating PI3K/AKT/mTOR and EERK1/2 pathways to promote proliferation, migration, invasion, and vascular mimetic formation (VM) in CRC [85]. LINC01559/miR-106b-5p/PTEN axis [174], and maturation of pri-miR-196b [175] and pri-miR-4717 [176] are regulated by the m6A modification of METTL3, these processes promotes hepatopulmonary metastasis and proliferation of cancer cells in CRC. In hepatocellular carcinoma, METTL3 increases m6A modification on SOCS2 mRNA, inducing SOCS2 suppression [177] and pri-miR-589-5p maturation, upregulating miR-589-5p expression, and promoting hepatocellular carcinoma cell migration and invasion [86]. Additionally, METTL3 is positively correlated with vasculogenic mimicry (VM) in hepatocellular carcinoma, the m6A modification of METTL3 might have enhanced the translation of YAP1, activating hippo signaling to induce vasculogenic mimicry in hepatocellular carcinoma and promoting metastasis [178]. METTL3 increases the m6A modification on anti-apoptotic Survivin mRNA, promoting its expression, inhibiting the cleavage activation of apoptotic execution protein Caspase3 and PARP, and enhancing hepatocellular carcinoma cell migration and invasion [179]. In intrahepatic cholangiocarcinoma (ICC), high expression of METTL3 mediates m6A modification of IFIT2 mRNA, declining IFIT2 mRNA in a YTHDF2 recognition-dependent manner, and promoting ICC proliferation and inhibiting apoptosis [88].

In non-small cell lung cancer (NSCLC), METTL3 adds YTHDF1/3 and eukaryotic translation initiation factor 3 subunit B (eIF3b) to the YAP mRNA translation initiation region [102], elevating m6A modification of circKRT17, enhancing YAP1 by recruiting EIF4 stability [180], promoting YAP expression and activity, inducing NSCLC metastasis [102], and inducing acquired resistance to osimertinib in lung adenocarcinoma (LUAD) [180]. Smoking is one of the causative factors of non-small cell lung cancer, METTL3 is upregulated and tumor suppressor death-associated protein kinase 2 (DAPK2) mRNA is degraded by m6A modification and YTHDF2 in NSCLC caused by smoking, so DAPK2 activates the NF-κB pathway and enhances tumor cell proliferation and migration [181]. METTL3-mediated m6A modification inhibits the H2A histone family member X (H2AX) mRNA degradation, enhancing DNA repair and cell survival, and promoting NSCLC cell proliferation, migration, and invasion [101].

In androgen treatment-sensitive prostate cancer, METTL3 mediates m6A modification of USP4 mRNA at A2696, and m6A reader protein YTHDF2 binds to and induces degradation of USP4 mRNA, increasing the expression of Rho GDP dissociation inhibitor alpha (ARHGDIA) protein and promoting migration and invasion [182]. METTL3 also inhibits the expression of growth inhibitor NKX3.1, promoting tumor cell proliferation [183]. METTL3 ectopic high expression in the cytoplasm promotes cell proliferation, invasion, and migration [24]. METTL3/14 complex mediates the m6A methylation of NAP1L2 mRNA, and LncRNA NAP1L6 recruits HNRNPC to the m6A sites of NAP1L2, promoting NAP1L2 mRNA stabilization, inducing cell migration, invasion, and enhancing EMT function [184]. METTL3 inhibits PTEN expression through the m6A-YTHDF2 modality and promotes prostate hyperplasia [185].

In breast cancer, the m6A modification-dependent effect of METTL3/YTHDF1/eEF1 increases the translation of KRT7 and promotes lung metastasis [186]. Zinc finger protein 217 (ZNF217) together with METTL3 increases the expression level of Nanog homeobox (NANOGA) through m6A modification and promotes BC cell EMT and invasion, but miR-135 hinders this malignant progression by inhibiting ZNF217 expression [187]. In thyroid cancer (TC), METTL3 regulates the maturation of miRNA-222 precursor pri-mRNA-222, and in turn, the mature miRNA-222 inhibits serine/threonine stress kinase 4 (STK4) expression as a sponge, promoting malignant cell proliferation and metastasis in TC [188]. In glioblastoma, the negative regulator of NF-κB, UBX domain protein1 (UBXN1), is degraded in response to m6A modification by METTL3, so increasing the NF-κB pathway activity and enhancing proliferation and migration of IDH wild-type glioma cells [189]. METTL3 and YTHDF1 proteins, abundantly express in glioblastoma, increasing the expression level of the RNA editing protein adenosine deaminase RNA specific (ADAR1), ADAR1 increases CDK2 expression level through its RNA-binding domain (RBD) to promote glioblastoma proliferation [110]. However, Wu et al. showed that METTL3 expression was downregulated in glioma, and METTL3 overexpression inhibited glioma malignant proliferation in vivo through circRNA SLC1/miR-671-5P/Beta-catenin-interacting protein 1 (CTNNBIP1) axis [108]. These two different findings may have been due to different degrees of malignancy and heterogeneity of gliomas. Further, the methyltransferase activity of METTL3 promotes glioblastoma stem cells (GSCs) proliferation, clonogenic capacity, and maintenance of tumor stemness. GSCs contribute to the tumor resistance to therapy, neoangiogenic tumor invasion, and immune escape [144]. High expression of METTL3 protein in SHH-medulloblastoma decreases PTCH1RNA stability and increases GLI family zinc finger 2 (GLI2) transcription and translation levels through m6A, thereby activating the Sonic hedgehog pathway, inhibiting tumor cell apoptosis, and promoting proliferation and tumor progression [190]. Cell cycle dysregulation is closely related to carcinogenesis and uncontrolled tumor cell proliferation. CDC25B from the cell division cyclin 25 (CDC25) family is associated with the activation of cyclin-dependent kinases (CDK1)/cyclin B during G2/M transformation. In cervical cancer cells, METTL3 induces m6A of CDC25B mRNA, then m6A sites are recognized by YTHDF1, promoting CDC25B translation to regulate cell cycle progression, inducing cell proliferation, and enhancing tumorigenicity in vivo [191]. RAB2B, a member of the RAS gene family, is regulated by the elevated expression of METTL3 [192]. METTL3 also can increase the expression of LncRNA-FOXD2-AS1 by m6A, LncRNA-FOXD2-AS1 silences the growth inhibitor p21 expression and promotes cervical cancer cell proliferation [193]. IGF2BP2 recognizes m6A sites of the cathepsin L (CTSL) mRNA 5ʹ-UTR region, enhancing its mRNA stability and promoting metastasis [94]. Thioredoxin domain-containing protein 5 (TXNDC5) is mainly located in the endoplasmic reticulum, and dysregulation of TXNDC5 is linked to oxidative stress, cellular senescence, and cancer, and is often expressed abnormally in cancer tissues. METTL3 inhibits the endoplasmic reticulum stress-induced apoptosis and autophagy by upregulating TXNDC5 expression in cervical cancer (CC) cells [93]. In ovarian cancer, METTL3 suppresses cyclin G2 (CCNG2) and PTEN by regulating the expression of pri-miR-1246 and pri-miR-126-5p, which in turn promotes cell proliferation, migration and invasion [194, 195].

Overexpressed METTL3 in thymic epithelial tumors (TETs) induces translation and expression of c-MYC by increasing the m6A modification of LncRNA MALAT1 and promotes cell proliferation and clonogenicity [98]. METTL3 recruits YTHDF1 to m6A sites, enhancing mRNA stability and translation of SLC7A11, inhibiting iron death and promoting proliferation in lung adenocarcinoma (LUAD) cells [196]. Abnormally high expression of METTL3 after PM2.5 exposure increases m6A modification of BIRC5 and recognized by IGF2BP3 inducing the mRNA stability and promotes bladder cancer cell proliferation, colony formation, invasion, and migration [146]. In gallbladder cancer (GBC), METTL3 degrades tumor suppressor gene dual-specificity phosphatase 5 (DUSP5) mRNA in a YTHDF2-dependent manner, thereby promoting proliferation, migration, and invasion of gallbladder cancer cells, GCS-SD and NOZ [100]. In ESCC, METTL3 targets the IFIT2 and inhibited its expression, thereby promoting proliferation, migration, and invasion of ESCC cells [97]. In Renal cell carcinoma (RCC), METTL3 m6A modifies zinc finger protein 677 (ZNF667), and the modification positively regulates its translation and mRNA stability by binding to YTHDF1 and IGF2BP2. Then, ZNF667 suppresses its expression by binding to the promoter of the tumor suppressor CDKN3, promoting RCC tumor proliferation and progression [197].METTL3 in clear-cell renal cell carcinoma (ccRCC) promotes cancer cell migration, invasion, and viability through m6A modification of HERV-H LTR-associating 2 (HHLA2) [96]. METTL3 in multiple myeloma (MM) enhances pri-miR-27a-3p maturation and Yin Yang 1 (YY1) mRNA stability by m6A modification, promoting MM cell proliferation, cell stemness, and suppressing apoptosis in vitro and in vivo [89]. The m6A modification of LncRNA DANCR by METTL3 increases its stability by regulating DANCR expression and thereby promotes OS cell proliferation, invasion, and migration [90]. In addition to upregulating histone deacetylase 5 (HDAC5) expression in OS cells, it promoted OS cell proliferation via the HDAC5/miR-142-5P/armadillo-repeat-containing 8 (ARMC8) axis [91] (Table 3).

### METTL14

Recently, Zhang et al. investigated 348 cholangiocarcinoma (CCA) patients, including 87 perihilar cholangiocarcinoma (pCCA) cases and 261 intrahepatic cholangiocarcinoma (iCCA) cases, and systemically compared the genetic alterations to demonstrate the tumor suppressive function of METTL14. METTL14 is a potential driver gene with recurrent and deleterious mutations in pCCA tumors. METTL14 is highly downregulated in pCCA tumors, its loss-of-function mutation R298H is deleterious that disrupting the m6A modification and increasing tumor proliferation and lung metastasis. Microtubule actin crosslinking factor 1 (MACF1), an oncogene involves in tumor metastasis, is identified as a downstream target of METTL14. The loss-of-function mutation R298H in METTL14 impairs its ability to mediate MACF1 degradation, leading to an increase in MACF1 expression [198].

SOX4 is identified as a downstream target of m6A METTL14, upregulation of SOX4 by METTL14 Knockdown enhances cancer metastasis via EMT and the PI3K/AKT pathway, dependent on YTHDF2 recognition [124]. Similarly, with the involvement of the reader protein YTHDF2, METTL14 modifies lncRNA XIST, which behaves as an oncogene in a variety of tumors, in an m6A-dependent manner [123]. Other reader proteins also mediate the tumor suppressive effect of METTL14 in colorectal cancer. In the presence of IGF2BP2, METTL14 targets tumor suppressor Kruppel-like factor 4 (KLF4) and degrades it via m6A modification, facilitating tumor metastasis [199]. ARRDC4 is also identified as a downstream target of METTL14 in colorectal cancer, and treatment with siRNAs against ARRDC4 significantly reverses the tumor migration, invasion, and metastasis induced by METTL14 knockdown [155]. METTL14 expression is down-regulated in ccRCC. The PTEN is an m6A target of METTL14, and YTHDF1 is responsible for maintaining the PTEN mRNA stability [200]. Integrin 4 (ITGB4) has also been designated as a METTL14 m6A modification target in ccRCC, ITGB4 expression is inhibited by METTL14, resulting in a tumor suppressive effect to attenuate the EMT process and deactivate PI3K/AKT signal pathway [201]. Recent elucidation reveals a positive feedback loop between METTL14 and ubiquitin-specific peptidase 38 (USP38). In bladder cancer, USP38 is a target of METTL14-mediated m6A modification. The stabilization of USP38 mRNA by METTL14 inhibits tumor migration, invasion, and EMT. USP38 in turn enhances the protein stability of METTL14 by promoting its deubiquitination in bladder cancer cells, further inhibiting bladder cancer development [202]. Another study identified METTL14 as a tumor suppressor in bladder cancer. Knockdown of METTL14 results in malfunction of the methyltransferase complex, reduction in tumor cell invasion and migration, and increase in apoptosis [203].

In gastric cancer, METTL14 high expression remarkably represses cancer cell growth and invasion. A circular RNA circORC5 acts as a sponge for miR-30c-2-3p and is identified as an m6A modification target of METTL14. METTL14 decreases circORC5 expression in an m6A-dependent way, upregulating miR-30c-2-3p and inhibiting the development of gastric cancer through the downregulation of AKT1S1 and EIF4AB [204]. A positive feedback loop consists of miR-99a‐5p, which yields tumor suppressive effects in ESCC. METTL14 is downregulated in ESCC and inhibits tribble 2 (TRIB2) expression through degradation of TRIB2 mRNA, targeting its 3′ untranslated regions. In turn, TRIB2 downregulates METTL14 by ubiquitin-mediated proteasomal degradation [205]. The miR-375 is a downstream target of METTL14, METTL14 reduces its expression by m6A modification and further decreases the SOX12 expression, mediating a cancer suppressive effect in breast cancer [206]. LncRNAOIP5-AS1, has been proposed as a METTL14 target in papillary thyroid cancer (PTC). METTL14 inhibits PTC cell proliferation and migration by direct binding to LncRNAOIP5-AS1 and activating the EGFR, AKT, and MEK/ERK signaling pathways [207].

Meanwhile, several pieces of evidence show METTL14 as an oncogene with pro-tumorigenic effects. Wang et al. reported that m6A levels were raised in about 70% of pancreatic cancer cases, and METTL14 was a major enzyme that modulated m6A methylation in pancreatic cancer. Through m6A modification of the PERP mRNA, over-expressed METTL14 significantly increases pancreatic cancer cell proliferation and migration [208]. METTL14 upregulation is linked to the poor prognosis in prostate cancer, mediating by the m6A downstream target thrombospondin 1 (THBS1) in YTHDF2 dependent manner [209].

In hepatocellular carcinoma (HCC), METTL14 targets hepatocyte nuclear factor 3*γ* (HNF3*γ*), and its expression is downregulated, and its stability is decreased m6A dependently, resulting in defective differentiation, resistance to sorafenib treatment [210]. LHPP, a histidine phosphatase and putative tumor suppressor gene, is downregulated in gastric cancer, possibly due to METTL14. As a consequence of GSK3b phosphorylation, HIF1A-involved glycolysis, proliferation, and metastasis in gastric cancer cells, METTL14 inhibits LHPP expression by m6A modification [211]. The m6A levels in HCC cells are upregulated after sublethal heat treatment, resulting in an upregulation of the E3 ligase neuronal precursor cell-expressed developmentally downregulates NEDD4. NEDD4 promotes tumor metastasis and growth through the TGF-*β* signaling pathway and directly binding to the TGF-*β* type I receptor (TGFBR1). METTL14 is the methyltransferase possibly responsible for this process [212]. ARHGAP5-AS1, a lncRNA, is an m6A target of METTL14 in HCC and promotes HCC development by inhibiting interactions between CSDE1 and TRIM28, hence degrading CSDE1 [212]. In the CCLE database, METTL14 expression is positively correlated to the genes of the cell cycle, DNA replication, and DNA damage repair in breast cancer. METTL14 knockdown likely increases the susceptibility to adriamycin, cisplatin, and olaparib, and decreased DNA replication and damage repair [213]. LNCAROD, an oncogenic lncRNA, is overexpressed in the head and neck squamous cell carcinoma (HNSCC) through METTL14-mediated m6A modification, improving its stability and advancing the T stage and poor prognosis [214] (Table 4).

## METTLs in reprogramming tumor cell metabolism

### METTL3

METTL3 participates in tumor metabolic reprogramming, and several studies on the regulation of lipid metabolism and glucose metabolism in tumor cells by METTL3 exist. Non-alcoholic fatty liver disease (NAFLAD) is the most common chronic liver condition worldwide, with a high prevalence in developed countries. Additionally, non-alcoholic fatty liver disease (NAFLD) can progress to non-alcoholic steatohepatitis (NASH), leading to a series of liver dysfunction and even liver cancer. Abnormal lipid metabolism contributes to the progression of NAFLD to liver cancer, and ATP citrate lyase (ACLY), an important enzyme in lipid formation, and SCD1, a rate-limiting enzyme in the monounsaturated fatty acid synthesis, are overexpressed in a mouse model of NAFLD. METTL3 and METTL14 stabilize mRNA of ACLY and SCD1 by m6A modification, and increases protein expression, thereby exacerbating triglyceride and cholesterol production and lipid droplet accumulation, promoting chronic inflammation, apoptosis, DNA damage, and excessive liver cell proliferation, and promoting the progression of NAFLD to HCC [215]. Rubicon is an autophagy inhibitory protein and upregulated METTL3 in NAFLD together with YTHDF1 modified RUBICON mRNA, promoting its stability. Subsequently, Rubicon inhibits autophagy and blocks the clearance of lipid droplets (LDs). Excessive lipid accumulation in hepatocytes is one of the triggers for the progression of NAFLD to HCC [216]. In addition to the m6A mechanism, nuclear METTL3 in non-alcoholic steatohepatitis (NASH) is transferred to the cytoplasm by phosphorylation of CDK9, causing the inability for METTL3 to directly regulate the promoters of CD36 and CCL2, thereby inhibiting their expressions, which promotes free fatty acid uptake, inflammation, and the progression of NASH [217]. The pro-oncogene stomatin-like protein 2 (SLP2) in hepatocellular carcinoma is regulated by METTL3-m6A-YTHDF1 with elevated expression levels, which interacts with JNK2 to promote the translocation of cytoplasmic AREBP1 to the nucleus, regulating lipid reprogramming and promoting lipid metabolism, especially lipid synthesis, in HCC cells [87]. Exploring the mechanism of METTL3 in lipid metabolism may find new directions in identifying a treatment approach for NASH-induced hepatocellular carcinoma in the future.

Normal cells obtain energy from aerobic oxidation; however, tumor cells have strong proliferation ability. To adapt to the change in tumor environment and growth status, tumor cells obtain energy differently. Even under aerobic conditions, they obtain more metabolites to maintain their rapid proliferation and select glycolysis for metabolism. This phenomenon is called the "Warburg effect". High expression of METTL3 and IGF2BP3 in gastric cancer mediates the m6A modification of HDGF mRNA to improve its stability and translation, and nuclear HDGF activates the expression of GLUT4 and ENO2 to increase the glycolysis of cancer cells and promote tumor growth and liver metastasis [139]. The expression of NADH dehydrogenase (ubiquinone) 1 alpha subcomplex 4 (NUDFA4), which is highly expressed and associated with poor prognosis in GC, promoting tumor cell glycolysis and oxidative metabolism [218]. In colorectal cancer, METTL3 increases the m6A modification of GLUT1 and HK2 mRNAs and enhanced mRNA stability and expression depending on the methylated IGF2BP2 and IGF2BP3, promoting the "Warburg effect" in tumors [219]. Furthermore, METTL3 promotes the "Warburg effect" in colorectal cancer through HIF-1*α* [220]. METTL3 may have increased HIF-1*α* mRNA through m6A-IGF2BP3. HIF-1*α* mRNA translation, in turn, promotes the transcription and translation of lactate dehydrogenase A (LDHA) using the combined effect of METTL3-m6A-YTHDF1. LDHA catalyzes the conversion of pyruvate to lactate, enhancing glycolysis in tumor cells [221]. Additionally, cytoprotective autophagy enables cancer cells to tolerate a hypoxic environment and promote cell survival and adaptation, and interactions between HIF-1*α* and the lncRNADARS-AS1 promoter promotes its transcription. LncRNA DAR-AS1 recruits METTL3/METTL14 to promote DARS translation and regulate hypoxia-induced cytoprotective autophagy in CC [222]. Lung adenocarcinoma (LUAD) accounts for approximately 60% of the incidence of non-small cell lung cancer. In LUAD, METTL3 and YTHDF1 enhance the translation of ENO1 by m6A modification to increase the glycolysis in tumor cells [223]. METTL3 increases m6A modification of ACLY and a mitochondrial citrate transporter SLC25A1, which is recognized by m6A readers IGF2BP2 and IGF2BP3 enhancing the mRNA stability, further affecting glycolytic pathway and promoting cell proliferation [224].

### METTL14

In a recent study by Liu et al., [225, 226] both wild-type METTL14 and the R298P mutant, which lacks the ability to mediate m6A modification, rescued the SASP-related genomic changes generated by METTL14 knockdown. The tumor-promoting function of SASP is mediated by METTL14 through its redistribution to the SASP gene enhancers in an m6A-independent manner. A high-fat diet is a risk factor for NASH and TGF-*β*1 is an essential pro-fibrogenic factor that mediates the transformation of NASH to fibrosis in hepatocellular carcinoma. In a rat model of NASH, METTL14 is activated by NF-B p65, leading to m6A modification on the 5′ untranslated region (UTR) of TGF-*β*1 mRNA, and resulting in an increased secretion of TGF-*β*1 and an accelerated development of fibrosis from NASH [227]. In a different study, an established NAFLD mouse model based on type 2 diabetes mellitus (DM2) showed spontaneous progression to NASH, fibrosis, and HCC. Comparable to human HCC, the NAFLD mice model exhibits an enhanced METTL14-mediated m6A modification in ATP citrate lyase (ACLY) and stearoyl-CoA desaturase1 (SCD1), resulting in overexpression of these two genes. Overexpression of METTL14 produces triglycerides and cholesterol, accumulating lipid droplets, which are directly associated with the development of NASH and HCC [228]. Specific to lipid metabolism, METTL14 is overexpressed and served as a dominant m6A-related enzyme in bladder cancer. Its m6A modification capability upregulates lncRNA DBET, which promotes the lipid metabolism of cancer cells by direct contact with FABP5 and activation of the PPAR signaling pathway. The METTL14/lncRNA DBET/FABP5 axis generated an oncogenic mechanism, mediating the development of bladder cancer [229]. Glycolytic reprogramming is strongly associated with tumor growth, angiopoiesis, and metastasis. In RCC, METTL14 is downregulated and bromodomain PHD finger transcription factor (BPTF) is upregulated through the enhanced stability associated with the decreased m6A modification in metastatic lesions. BPTF subsequently activates downstream targets enolase 2 (ENO2) and SRC proto-oncogene (SRC) to initiate the glycolytic reprogramming associated with the metastasis in RCC cells [230]. Similarly, in hepatocellular carcinoma, METTL14 plays critical roles in glycolytic reprogramming by regulating the ubiquitin-specific peptidase 48 (USP48) mRNA stability. METTL14 acts as a tumor suppressor to attenuate glycolysis and malignancy in HCC through the Mettl14-USP48-SIRT6 axis [231].

## METTLs in tumor immune response

### METTL1

Radiofrequency ablation (RFA) is currently a primary treatment for early-stage HCC, and patients benefit from the RFA due to its minimally invasive nature and safety, but like other treatments its relapse rate is high. The advantage of RAF-inducing anti-tumor immunity is diminished by the tumor immunosuppressive effect of the aggregates of MDSCs in the tumor microenvironment. Insufficient radiofrequency ablation (iRFA) enhances the expression of METTL1 in recurrent HCC tissues, forming a tumor immunosuppressive microenvironment through the METTL1-TGF-*β*2-PMN-MDSC axis, increasing polymorphonuclear-myeloid derived suppressor cells (PMN-MDSCs) infiltration, and downregulation of METTL1 or blockade of TGF-*β* signaling, significantly alleviating iRFA-induced HCC tumor progression [75]. METTL1 promotes infiltration of PMN-MDSCs in human TIMs by regulating Interleukin-8 (CXCL8) that inhibits the effectiveness of PD-1 treatment. Blocking METTL1 and its downstream chemotactic pathway effectively enhances the anti-PD-1 efficacy [232]. High expression of METTL1/WDR4 in nasopharyngeal carcinoma (NPC) mediates m7G tRNAs modification, upregulating WNT/*β*-catenin pathway, and promoting epithelial-mesenchymal transition (EMT) and chemoresistance to cisplatin and docetaxel in NPC cells [80].

### METTL3

Under normal circumstances, immune surveillance can recognize and remove mutant cells from the body, maintaining a homeostasis of the immune internal environment and preventing tumorigenesis. However, tumor cells can generate a series of self-protection mechanisms against the body's immunity and survive various stages of anti-tumor immunity. Recent studies found the role of METTL3 in regulating PD-L1 expression in tumor cells and thus influencing tumor immune response. METTL3 mediates m6A methylation of circIGF2BP3 in NSCLC. YTHDF1 recognition increases its expression in a manner that promotes its post-transcriptional splicing. Subsequently, circIGF2BP3 stabilizes the downstream OTUB1 mRNA, and increased expression of the deubiquitinating enzyme OTUB1 regulates the ubiquitination of the immune checkpoint protein PD-L1, inhibiting the degradation of PD-L1 in the endoplasmic reticulum, and evading the killing by CD8^+^ T cells, and causing immune escape of tumor cells [233, 234]. In bladder cancer, PD-L1 is regulated by the m6A modification, its RNA is more stable, and translation and expression are elevated in the presence of METTL3 and IGF2BP1, which help bladder cancer resist the cytotoxic effects of CD8^+^ T cells [147]. METTL3-IGF2BP3 increases the PD-L1 expression on breast cancer cell membranes using an m6A modification recognition to mediate immune escape [235]. The methyltransferases METTL3/METTL14 modulate the immune response to anti-PD-L1 therapy, and the knockdown of METTL3 inhibits m6A modification, enhancing the sensitivity to PD-1 therapy in pMMR-MSI-L colorectal cancer and melanoma patients [236]. The PD-L1 expression is significantly increased in cervical cancer and positively correlated to METTL3 expression [237]. METTL3 can be a potential target for immunotherapy. In animal experiments, STM2457, a small molecule inhibitor targeting METTL3, enhancing the therapeutic effect of anti-PD1, and the combination of the two show good anti-tumor efficacy in colorectal cancer [238]. Similarly, highly-expressed METTL3 in cervical squamous cell carcinoma (CESC) is negatively correlated to tumor immune cell infiltration and promotes the expression of immune checkpoint molecules (PD-L1). The combination of anti-PD-L1 therapy and METTL3 inhibitor promote immunotherapy in vivo in CESC [239].

Malignant tumors are immunosuppressed, and the tumor microenvironment (TME) accumulates a large number of tumor-infiltrating myeloid cells (TIMs), including tumor-associated neutrophils (TANs), tumor-associated macrophages (TAMs), regulatory dendritic cells (DCregs), and myeloid-derived suppressor cells (MDSCs), that aid tumors resist treatment and participate in immune escape. Growth factors, chemokines, and pro-inflammatory cytokines in TME coordinate the function and differentiation of TIMs [240]. TAMs are important for innate immunity in the tumor microenvironment. Classically activates M1 macrophages have anti-tumor activity, while alternatively activates M2 macrophages promote tumor growth and metastasis by suppressing inflammation and inducing angiogenesis in the tumor microenvironment. A small lipid molecule lipoxin A4 (LXA4) secreted by prostate cancer cells downregulates METTL3 expression in macrophages in the tumor microenvironment, which in turn activates STAT6, promoting macrophage differentiation to M2 type, suppressing immune responses in the tumor microenvironment, and supporting tumor growth and metastasis [241]. Lactate in TEM mediates METTL3 K281 and K345 lactonization, upregulating METTL3 in colon cancer infiltrating TIMs, and upregulates METTL3 in TIMs mediates m6A modification on Jak1 mRNA. The m6A-YTHDF1 axis enhances the Jak1 protein translation and subsequent STAT3 phosphorylation, promoting immunosuppressive gene transcription and enhancing immunosuppression in TIMs [242]. METTL3 is downregulated in papillary thyroid tumors and synergized with YTHDF2 to promote inflammatory cytokine IL-8 secretion through activation of the NF-κB pathway. The IL-8 promotes TANs activation and TANs in the tumor microenvironment are associated with poor tumor prognosis. The CCL4^+^ PD-L1^+^ TANs recruit macrophages, inhibited T cell cytotoxicity, showing immunosuppressive functions, and promoting tumor development [106, 243]. Low expression of METTL3 in papillary thyroid tumors and melanoma increases the infiltration of inflammatory cytokines IL-8, CXCL10, and CXCL9 in the tumor microenvironment [106, 236]. MDCSs strongly inhibits the anti-tumor activity of NK cells and T cells, thereby impairing anti-tumor immunity. METTL3 increases basic helix-loop-helix family member e41 (BHLHE41) expression in an m6A-dependent manner, which in turn promotes the CXCL1 transcription, induced MDSC migration around the tumor, and suppressed CD8^+^ T cell immune response to tumor cells. Silencing METTL3 in colorectal cancer (CRC) cells reduces the accumulation of MDCSs, increasing CD4 ^+^ and CD8^+^ T cell immune function activation and proliferation, and inhibiting CRC development [238]. In the tumor microenvironment, METTL3 deficiency in NK cells inhibits its immune response, thereby promoting immunosuppression. [244].

### METTL14

METTL14 possibly plays a role in multiple aspects of anti-tumor immunity. In tumor-associated macrophages (TAMs), macrophage-specific deletion of METTL14 produces defective CD8^+^ T cell differentiation by affecting the transformation of intratumoral CD8^+^ T cells, hence inhibiting the ability of CD8^+^ T cells to eliminate malignant cells. This macrophage^−^CD8^+^ T cell crosstalk is mediated by Epstein–Barr virus-induced protein 3 (Ebi3), a subunit of both IL-27 and IL-35 heterodimeric cytokines. Targeting Ebi3 in macrophage cells by neutralizing with Ebi3 antibody reduced tumor development and activating CD8^+^ T cells in mice [245]. In another study, over-expressed METTL14 effectively reduces tumor development in vitro and is accompanied by downregulation of TIR domain-containing adaptor molecule 2 (TICAM2) expression and toll-like receptor 4 (TLR4) suppression. METTL14 overexpression and TLR4 agonist suppress tumor development and promote macrophage M1 polarization. Cyclic (Arg-Gly-Asp) (cRGD) peptide-modified macrophage membrane-coated nanovesicles are constructed to co-deliver METTL14 and the TLR4 agonist. These nanovesicles are capable of targeting tumors and macrophages, reducing tumor development and triggering M1 polarization in macrophages [246].

METTL14 is an immune-related gene that positively correlates to immune infiltration and plays a role in immunotherapy [247, 248]. METTL14 possibly has a function in therapies using Immune checkpoint blockade (ICB). In colorectal cancer, the depletion of METTL14 in tumor cells improve the effectiveness of anti-PD-1 therapy [247]. METTL14-deficient tumors cause an increase in tumor-infiltrating CD8^+^ T cells in vivo and an immune-activated milieu is characterized by enhanced production of IFN-, Cxcl9, and Cxcl10. IFN-*γ*-Stat1-Irf1 signaling is activated during this process, contributing to increase stability of Stat1 and Irf1 mRNA [236]. Additionally, METTL14 regulates ICB immunotherapy efficacy in cholangiocarcinoma through m6A modification in the 3'UTR region of the seven in absentia homolog 2 (Siah2) mRNA and subsequent degradation via YTHDF2. Siah2 is a RING E3 ubiquitin ligase capable of promoting PD-L1 K63-linked ubiquitination. High expression of METTL14 may be a biomarker of ICB immunotherapy sensitivity [249]. In hepatocellular carcinoma, METTL14 promotes m6A modification of MIR155HG and then stabilizes it in a HuR-dependent manner. As a ceRNA, MIR155HG can regulate the PD-L1 expression through miR-223/STAT1 axis. METTL14 and MIR155HG might be ICB immuno-therapeutic targets [249].

## METTLs in tumor chemotherapy

### METTL1

Lenvatinib, a tyrosinase inhibitor, is used in the treatment of advanced hepatocellular carcinoma as a first-line drug, but the chemoresistance seriously affects its efficacy. In proteomic assays of lenvatinib-resistant cell lines, METTL1/WDR4 mediate m7G modification and is highly expressed in cells. METTL1/WDR4-modified m7G tRNA promotes the translation of EGFR pathway genes, promoting HCC lenvatinib resistance. Using METTL1/WDR4 as a resistance marker or intervention target will provide a promising direction to address the chemotherapy resistance [250].

### METTL3

Chemoresistance by tumors leads to clinical treatment failure, causing an important challenge in the fight against tumors. Previous studies found that epigenetic modifications were involved in the mechanism of chemoresistance. METTL3 and YTHDF1 co-m6A modify FOXO3 mRNA and increase their stability to promote FOXO3 expression. However, in sorafenib-resistant hepatocellular carcinoma cells, METTL3 expression is decreased, m6A modification of FOXO3 is reduced, and the degradation is increased, inducing sorafenib resistance by activating the autophagic pathway [251]. CircVMP1 is upregulated in the serum exosomes in non-small cell lung cancer, and METTL3 and SOX2 expression is upregulated through the miR-524-5p/METTL3/SOX2 axis, promoting cancer cell proliferation, apoptosis inhibition, and cisplatin resistance [252]. METTL3 is highly expressed in lung adenocarcinoma tissues and mediates m6A modification to upregulate the lncRNA SNHG17 expression. The upregulated SNHG17 inhibits its expression by recruiting EZH2 to the large tumor suppressor kinase 2 (LATS2) promoter region, promoting LUAD progression through the METTL3/SNHG17/LATS2 axis [253], and is involved in gefitinib resistance. *β*-Elemene can inhibit resistance by directly targeting the *S*-adenosylmethionine-binding domain of METTL3 to inhibit its methyltransferase activity [103]. METTL3-YTHDC1 mediates chemoresistance in breast cancer patients during the adriamycin treatment by increasing m6A modification of EGF mRNA, upregulating the expression level of RAD51, and improving the homologous recombination repair (HR) efficiency [254]. METTL3 protein level is significantly increased in breast cancer tamoxifen-resistant (TamR) MCF-7 cells, increased m6A modification of AK4 stimulating ROS and p38 phosphorylation, and inhibiting mitochondrial apoptosis to produce chemoresistance [255]. BRAF^V600E^ mutation-positive advanced melanoma is treated with the Raf inhibitor PLX4032, however, METTL3 expression is upregulated in PLX4032-resistant cells, increasing m6A modification on EGFR mRNA, enhancing its translation, activating the RAF/MEK/ERK pathway, and mediating the onset of drug resistance [256]. Oxaliplatin is a first-line chemotherapeutic agent for the treatment of advanced gastric cancer and CD33^+^ gastric cancer stem cells are the main subpopulation of oxaliplatin resistance in gastric cancer. PARP1 effectively restores the DNA damage caused by oxaliplatin by mediating the base excision repair pathway. It is an important gene leading to drug resistance in CD33^+^ stem cells with high METTL3 expression and utilizes m6A modification to regulate PARP1 stability and expression, thereby promoting oxaliplatin resistance in CD33^+^ gastric cancer stem cells [257]. A lncRNA that binds to apoptotic protease-activating factor 1 (APAF1) ABL is regulated by elevated expression of METTL3-m6A-IGF2BP1. ABL binds to APAF1 to inhibit its function thereby preventing caspase 9/3 activation to inhibit apoptosis and promote GC cell survival and multidrug resistance [258]. METTL3 is highly expressed in 5-fluorouracil (5-FU)-resistant colorectal cancer cell lines, and GLUT and HKs are elevated, inducing drug resistance through enhanced glycolysis [219, 221, 259]. Further, elevated METTL3 promotes the 5-FU resistance by regulating RAD51-associated protein 1 (RAD51AP1) expression [260]. Targeting METTL3 may be a breakthrough to overcome 5-FU resistance in colorectal cancer. Intermediate filament family orphan 1 (IFFO1) is a novel tumor suppressor that inhibits ovarian cancer metastasis and cisplatin resistance. However, histone deacetylase (HDAC5)/YY1 represses IFFO1 transcription in ovarian cancer, while METTL3-m6A-YTHDF2 induces its mRNA degradation. The IFFO1 expression at both transcriptional and post-transcriptional levels is suppressed, resulting in tumor development, metastasis, and cisplatin resistance [261]. Highly expressed METTL3 in AML increases the m6A of MYC to enhance its expression and promoted arabinocytosine (AraC) chemoresistance [150]. Highly expressed METTL3 in glioma microvascular ECs (GECs) promotes m6A of cytoplasmic polyadenylation element-binding protein 2 (CPEB2) mRNA methylation, is enhanced by IGF2BP3 recognition, CPEB2 induces serine and arginine rich splicing factor 5 (SRSF5) mRNA stability and mediated selective splicing of transcription factor ETS1. Subsequently, it regulates the levels of tight junction-related proteins ZO-1, occludin, and claudin-5. The cellular bypass pathway knockdown of METTL3 increases the permeability of BTB and promotes drug penetration, improving the efficacy of glioma chemotherapy [112].

### METTL7A/METTL7B

METTL7A and METTL7B belong to the methyltransferase family, and the interaction of tumor cells with surrounding cells can induce malignant features of tumors. Multiple myeloma (MM) cells enhance METTL7A activity in surrounding adipocytes through the EZH2-mediated protein methylation. METTL7A in adipocytes uses its methyltransferase activity to lncRNA LOC606724 and lncRNA SNHG1 for m6A methylation modification, increasing their stability to release into exosomes. These lncRNAs induce apoptosis in MM cells to produce chemoresistance [262]. Methotrexate (MTX) is commonly used in the treatment of choriocarcinoma, but a significant proportion of patients are chemoresistant to MTX. METTL7A and DDP4 expression are increased in the MTX-resistant cell lines. Activation of PI3K/AKT, ERK1/2, and STAT3 pathways inhibit apoptosis in choriocarcinoma cells and promoted MTX resistance [263]. In most NSCLCs, METTL7B promotes the NSCLC cell proliferation by regulating the cell cycle-related gene CCND1 [264]. Highly expressed METTL7B in cell lines resistant to EGFR-TKIs relies on its methyltransferase activity to increase the level of m6A modification of glutathione peroxidase 4 (GPX4), heme oxygenase 1 (HMOX1), and superoxide dismutase 1 (SOD1), increasing protein expression and enzyme activity, inducing LUAD on gefitinib and osimertinib resistance [6]. The use of METTL7B as a potential therapeutic target can improve the efficacy of EGFR inhibitors. METTL7B expression is also significantly higher in LUAD cancer tissues than in the paracancer tissues and is mainly distributed in the cytoplasm, significantly promoting LUAD metastasis [265]. RhoBTB1 and 2, members of the Rho GTPase family, regulating the expression of methyltransferases METTL7B and METTL7A, respectively; however, RhoBTB1 and 2 are often silent or mutate in a wide range of epithelial cell cancers, resulting in Golgi fragmentation-induced breast cancer cell invasion [266].

### METTL14

In renal cell carcinoma, METTL14 mediates the sunitinib treatment resistance. METTL14 enhances the tumor necrosis factor receptor-associated factor 1 (TRAF1) RNA stability in an m6A-dependent manner. TRAF1 knockdown reverses the sunitinib resistance in tumor cells [267]. In osteosarcoma (OS), METTL14 mediates the all-trans-retinoic acid (ATRA) chemotherapy resistance through the m6A modification in the CDSregions of the meningioma 1 (MN1), an ATRA resistance-related gene in AML patients. IGF2BP2 was the reader protein of the m6A-marked MN1 mRNA, preventing its degradation, and enhancing its efficiency of translation [268]. METTL14 is overexpressed in pancreatic cancer, and elevated METTL14 levels facilitated cisplatin resistance. The mTOR signaling pathways re-sensitized the pancreatic cancer cells to cisplatin after the siRNA-mediated reduction of METTL14 [269]. DDP is also widely used in non-small cell lung cancer (NSCLC) as first-line standard chemotherapy. The DDP resistance is mediated by METTL14 through the miR-19a-5p/RBM24/AXIN1 axis in NSCLC. In vivo METTL14 silencing reduces the amount of pri-miR-19a m6A and re-sensitized the NSCLC to DDP therapy [270]. E2F transcription factor 8 (E2F8) is a target of METTL14 m6A modification, and YTHDF1 influences the E2F8 mRNA stability, promoting resistance to DDP and PARP inhibitor olaparib in breast cancer cells [213]. The m6A modification represents an early adaptive response of cancer cells to oxidative stress generated by chemotherapeutic agents [271–273]. In a recent study on oxidative stress utilizing hydrogen peroxide (H2O2), oxidative stress enhanced METTL14-mediated m6A modification of pleckstrin homology-like domain, family B, and member 2 (PHLDB2) mRNA, hence enhancing its protein production. As a result, PHLDB2 upregulation stabilizes and promotes the nuclear translocation of the epidermal growth factor receptor (EGFR). Activated EGFR signaling induces resistance to cetuximab in latent CRC metastases [274].

## Diagnostic value and prognosis evaluation in cancer

Some members of the METTL family protein play a driving role in cancer development, and studies at the clinical histological level have found that the protein expression level of METTLs correlate with prognosis of patients, METTL1 is overexpressed in a variety of cancers, and its high expression is often associated with poor progression, high expression of METTL1/WDR4 is associated with advanced stages and poorer survival in HCC, NPC and ICC [74, 80, 167]. In bladder cancer, high expression of METTL1 is also associated with poor prognosis [76]. A pan-cancer analysis found that METTL1 has the potential to assess patient prognosis, and high METTL1 expression is positively correlated to tumor progression-related pathways [82]. The METTL1 has potential diagnostic and therapeutic values in many cancers and can be used in the future. The m7G tRNA of METTL1/WDR4 is upregulated in Head and neck squamous cell carcinoma (HNSCC) and associated with poor prognosis [77].

High METTL3 expression in tumor tissues is significantly associated with poor prognosis, clinical stage cancer, and distant metastasis [84, 86, 101, 223]. Further, GC patients with higher cytoplasm/nucleus ratio and higher cytoplasmic METTL3 expression has significantly worse overall survival, and prostate cancer patients have higher Gleason scores, suggesting that the cytoplasmic distribution of METTL3 can be used as a diagnostic indicator or can serve as a therapeutic target for tumors [24]. Upregulation of METTL3 is positively correlated with high malignancy and poor prognosis in IDH wile-type gliomas [111]. Analysis of clinical data shows higher levels of METTL3 and IGF2BP3 expression in tumor tissues in PD-1/PD-L1 treatment-sensitive patients [235]. However, we need to note that some studies shows METTL3 expression is low in glioma and patients with low-expression glioblastoma had shorter disease-free survival [275]. In addition this, stimulation of the human papillomavirus (HPV) contributes to squamous carcinogenesis to some extent, and METTL3 is correlated to HPV carcinogenicity. An analysis of 1485 patients with head squamous cell carcinoma (HNSC) and 507 patients with cervical squamous carcinoma (CESC) found that METTL3 is positively correlated to HPV-associated positive tests E6 and unspliced-E6 expression, and p16 expression is positively correlated to poor prognosis and suppressed tumor immune microenvironment in HPV-associated tumors [239].

METTL5 can be used with other m6A RNA methylation regulators as a reliable predictive factor to independently predict the prognosis of pediatric AML patients and the efficacy of chemotherapy and immunotherapy in SCLC patients [276–278]. METTL6 is significantly in HCC tissues and correlated to poor survival outcomes in HCC [42]. METTL7B affects the development of multiple tumors, and high expression in gliomas is linked to poor cancer prognosis [279]. METTL7B expression is upregulated in most NSCLCs and induced advanced tumor development, leading to reduced patient survival [264]. METTL13 exerts a pro-carcinogenic role in several cancers [118]; however, in renal clear cell carcinoma, patients with high METTL13 expression showed a good prognosis [118].

In colorectal cancer, METTL14 has a tumor-suppressive effect. The downregulation of METTL14 in colorectal cancer is correlated to poor prognosis [123, 124, 248]. METTL14 upregulation is linked to the poor prognosis in prostate cancer and HCC [209, 210]. A prognostic model m6A score is developed utilizing m6A-associated gene signature to predict the prognosis of metastatic prostate cancer, and high m6A scores are linked to a bad prognosis, METTL14 is a critical gene in the m6A score, and its overexpression promotes the invasion and metastasis of prostate cancer cells [280].

In gastric cancer and hepatocellular carcinoma, METTL16 is upregulated and its increased expression is associated with poor patient prognosis [126, 129]. Elevated expression of 17 m6A regulators, including METTL16 is seen in esophageal cancer [281], and METTL16 is highly expressed in breast cancer with low survival rates [282]. However, the different is that high METTL16 expression is positively correlated to overall survival in thyroid cancer and adrenocortical carcinoma [130], low expression predicts poor OS and DFS in HCC [131]. Multiple METTL proteins are highly expressed in tumor tissues and promotes cancer development. The combined analysis of methyltransferase expression predicts the patient’s prognosis and survival, these highly expressed METTL proteins can be used as potential targets in future tumor therapy [276–278]. However, it should be noted that METTLs may play a role in both promoting and suppressing cancer progression in highly heterogeneous tumors, which requires more studies in the future to explain this.

## Targeting METTLs using small molecular inhibitors

### METTL3

METTL3 regulates the expression and function of target genes by catalyzing m6A methylation modifications in several tumors. METTL3 has potential value as a new therapeutic target due to its pro-cancer role in many cancers. Designing small molecule inhibitors targeting the SAM-binding region to impede its catalytic ability or designing small molecules targeting the MTC complex to disrupt its functional structural scaffolding and impair its m6A modification function are concepts for the development of small molecule inhibitors. Quercetin derived from natural products can fill the pocket of the adenosine moiety of SAM and inhibit its methyltransferase activity [283], clinical trials have focused more on the anticancer effects of quercetin, the efficacy of improving patients' physical weakness, the relief of neuralgia caused by chemotherapy, and the effects of oral mucositis, and most of these clinical trials are in the phase II stage. Delicaflavone (DLL), a biocomponent extracted from Selaginella doederleinii Hieron inhibits METTL3/14 expression and activates an anti-tumor immune response to prevent lung cancer development [283]. The most studied small molecule inhibitor STM2457 effectively inhibits METTL3 expression in mice with impaired leukemic stem cell function and prevents AML development [284], effectively inhibits prostate cancer cell proliferation and invasion, and inhibits SHH-medulloblastoma progression [95, 190]. STM2457 inhibits the ICC cell proliferation in a dose-dependent manner, promoting ICC cell apoptosis, and arresting the ICC cell cycle in S phase [88]. Small molecule inhibitor STM2457 plus anti-PD-1 treatment exhibits good anti-tumor effects in colorectal cancer [238]. High-throughput docking METTL3 screened and validated adenine derivative 2 and 7 are developed as small molecule inhibitors of METTL3 [285]. CDIBA derivative display potent enzyme inhibitory activity of the METTL3-14 complex and an antiproliferative effect in the AML cell lines by suppressing the m6A level of mRNA [286]. Using a structure-based drug discovery approach, a METTL3 inhibitor, UZH1a, has been identified, and treatment with UZH1a results in reduced levels of m6A mRNAs, increasing apoptosis, and cell cycle arrest in cell lines [287]. UZH1a, a small molecule inhibitor targeting METTL3, effectively suppresses tumor volume in the glioblastoma PDX mice model, and the combination treatment with UZH1a controlled tumor growth and prolonged survival in mice compared to a single agent [144]. METTL3 and METTL14 form a complex to exert methyltransferase activity, and the metastable inhibitor Eltrombopag binds to the METTL3/14 complex to inhibit its activity, effectively reducing the level of m6A modification in AML cell lines and exhibiting anti-AML cell proliferation, aiding to develop new anti-AML therapeutic agents using METTL3/14 as a target [288], Eltrombopag is first reported as a thrombopoietin receptor (TPO-R) agonist for the treatment of immune thrombocytopenia, and in 2008 and 2014, the U.S. Drug and Food Administration approved its use for the treatment of chronic ITP and aplastic anemia, respectively [289–291]. However, METTL3-mediated m6A modification has an important regulatory role in the central and nervous systems [292], and therefore, the systemic use of METTL3 inhibitors remains to be further considered (Fig. 2B and Table 5).

In current studies, many small molecule inhibitors are designed for METTL3 or METTL3/14 complex, and there are fewer studies on small molecule inhibitors of other members of the METTLs protein family. M6A modification in which members of the METTLs family proteins play an important role in human cancers, so there are many studies on small-molecule inhibitors of m6A modifiers. For example, there are many studies on small molecule inhibitors of m6A demethylase FTO, and more than ten FTO inhibitors have been identified and evaluated for their therapeutic effects in vitro and vivo tumor models [293]. Inhibitors of FTO show a significant anti-tumor efficacy in glioma [294], AML [295], and melanoma [296]. In addition, other small molecule inhibitors of m6A modifiers have been found. For example, a small molecule inhibitor of ALKBH5, ALK-04, is found by using X-ray crystal structure in silico screening of compounds, and significantly inhibit melanoma growth in mice [297]. Compound screening identify that BTYNB has the ability to selectively inhibit IGF2BP1 protein and c-MYC [298]. These studies have shown the potential value of small molecule inhibitors of m6A modifiers.

## Conclusion and future directions

Epigenetic modifications encompass various complex mechanisms that regulate malignant tumor progression, with the methyltransferase-like protein family playing a critical role in RNA methylation. These proteins influence gene transcription and translation via methylation modification mechanisms, further regulating tumor cell proliferation, metastasis, metabolic reprogramming, and responsiveness to immunotherapy and chemotherapy. Therefore, understanding the function of the methyltransferase protein family is crucial for suppressing tumor progression and enhancing the clinical treatment efficacy of tumors.

Currently, the roles of METTL3 and METTL14 in tumors have been extensively investigated, with most research results suggesting that METTL3 acts as an oncogene. Furthermore, some small molecule inhibitors targeting the catalytic activity of the METTL3 and METTL14 methyltransferase complex have been developed. These drugs have demonstrated inhibitory effects on malignant tumor progression in cellular and animal experiments. However, some small molecule drugs remain in the early stages of development and require testing across a wider range of tumor types, with more preclinical research needed to explore their potential therapeutic value. Moreover, some methyltransferase proteins are involved in regulating stem cell differentiation, neuroembryonic development, and mitochondrial metabolism, depending on their methyl catalytic activity. Elucidating these regulatory mechanisms will further clarify tumor formation mechanisms and tumor cell metabolic reprogramming. Notably, METTL3 not only functions through its methyltransferase activity but can also promote transcript translation independent of its m6A modification mechanism and increase P53 mRNA stability [299, 300]. Nonetheless, there are limited studies in this area, and future research should also concentrate on the m6A-independent functions of the METTLs family, particularly the m6A-independent cancer-promoting functions.

**Table 3:** The role of METTL3 in various cancers

Role	Cancer	MTTL3 targets	Downstream	Prognosis*	Mechanism	Cellular function	References
Oncogene	Gastric cancer	HDGF	GLUT4, ENO2	Poor	RNA stability by IGF2BP3	Migration, proliferation	[139]
		ZMYM1	E-cadherin	Poor	RNA stability by HuR	EMT, migration	[169]
		SPHK2	KLF2	Poor	RNA translation by YTHDF1	Proliferation, migration, invasion	[170]
		PARP1		Poor	RNA stability by YTHDF1	Oxaliplatin resistance	[257]
		LncRNA THAP7-AS1	CUL4B	Poor	RNA stability by IGF2BP1	Growth, migration, and invasion	[171]
		NDUFA4		Poor	RNA stability by IGF2BP1	Proliferation, ROS, glycolysis	[218]
	Colorectal cancer	GLUT1	mTORC1	Poor	RNA translation	Proliferation	[219]
		GLUT1, HK2		Poor	RNA stability by IGF2BP2/3	Proliferation, glycolysis	[259]
		HIF-1*α*		Poor		Glycolysis	[220]
		Sox2	CCND1, MYC, POU5F1	Poor	RNA stability by IGF2BP1/2	Cell stemness, metastasis	[301]
		Sec62	Wnt/*β*-catenin		RNA stability by IGF2BP1	Cell stemness, chemoresistance	[302]
		CRB2	Hippo	Poor	RNA decay by YTHDF2	Proliferation, migration, invasion	[173]
		EphA2、VEGFA	PI3K/AKT/mTOR、EERK1/2	Poor	RNA stability by IGF2BP2/3	Proliferation, migration, invasion, VM	[85]
		LINC01559	miR-106b-5p/PTEN	Poor		Proliferation, metastasis	[174]
		pri-miR-196b	miR-196b	Poor		Metastasis	[175]
	Liver carcinoma	SOCS2		Poor	RNA decay by YTHDF2	Proliferation, migration	[177]
		YAP1	VE-cadherin, MMP2, MMP9	Poor		Vasculogenic mimicry	[178]
		ACLY, SCD1		Poor	RNA m6A modification by METTL14	Lipid metabolism	[215]
		Rubicon			RNA translation by YTHDF1	Autophagy	[216]
		FOXO3			RNA stability by YTHDF1	Autophagy, Chemoresistance	[251]
		SLC7A11			RNA stability by IGF2BP1	Ferroptosis resistance	[303]
		IFIT2		Poor	RNA decay by YTHDF2	Proliferation, invasion, apoptosis	[88]
		Pri-miR-589-5p	miR-589-5p	Poor		Malignant development	[86]
		HKDC1	JAK2/STAT1/caspase-3			progression	[304]
	Lung cancer	YAP		Poor	RNA translation by YTHDF1/3	Migration, chemoresistance	[102]
		circIGF2BP3	PKP3, OTUB1		RNA	Immune escape	[233]
		DAPK2	NF-*κ*B	Poor	RNA decay by YTHDF2	Proliferation, migration	[181]
		SOX2				Proliferation, migration, invasion, DDP-resistance	[252]
		ENO1		Poor	RNA translation by YTHDF1	Warburg effect	[223]
		SLC7A11		Poor	RNA stability and translation by YTHDF1	Proliferation, inhibits ferroptosis	[196]
		LncRNA SNHG17	LATS2	Poor		Migration, invasion, EMT, resistance	[253]
	Prostate cancer	USP4	ARHGDIA	poor	RNA decay by YTHDF2、HNRNPD	Invasion, migration	[182]
		NKX3-1, LHPP		Poor	RNA decay by YTHDF2	Proliferation, migration	[183]
	Bladder cancer	PD-L1		Poor	RNA stability by IGF2BP1	Immune escape	[147]
		BIRC5		Poor	RNA stability by IGF2BP3	Proliferation, migration, invasion	[146]
		pri-miR221/222		Poor	Pri-mRNA maturation by DGCR8	Proliferation	[20]
	Breast cancer	EGF	RAD51	Poor	RNA translation by YTHDC1	Chemoresistance	[254]
		KRT7			RNA stability by IGF2BP1/HuR RNA translation by YTHDF1/eEF1	Migration	[186]
		PD-L1		Poor	RNA stability by IGF2BP3	Immune escape	[235]
		AK4	p38, ROS	Poor	RNA stability by ?	Apoptosis, chemoresistance	[255]
	Thyroid carcinoma	c-Rel	IL-8		RNA decay by YTHDF2	Proliferation	[106]
		pri-miR-222-3p	STK4		promote pri-miRNA to mature	Proliferation, migration, invasion	[188]
	Chronic myeloid leukemia	PES1, MYC		Poor	RNA m6A modification by METTL14	Proliferation, imatinib resistant	[299]
	Glioblastoma	ADAR1	CDK2	Poor	RNA stability by YTHDF1	Proliferation	[110]
		LncRNA MALAT1	NF-κB	Poor	RNA stability by HuR	Proliferation, malignancy	[111]
		UBXN1	NF-κB	Poor	RNA decay by YTHDF2	Proliferation, migration	[189]
	Medulloblastoma	PTCH1, GLI2	Sonic hedgehog signaling	Poor		Proliferation	[190]
	Melanoma	EGFR	RAF/MEK/ERK	Poor	RNA translation by ?	Chemoresistance	[256]
		PD-L1, Stat1, Irf1			RNA decay by YTHDF2	IMMUNE escape	[236]
	Cervical cancer	HK2		Poor	RNA stability by YTHDF1	Proliferation, Warburg effect	[305]
		LncRNA FOXD2-AS1	p21			Proliferation, migration, apoptosis	[193]
		RAB2B		Poor	RNA stability by IGF2BP3	Proliferation	[192]
		TXNDC5	ER stress	Poor	YTHDF1/2、IGF2BP2/3	PROLIFERATION, migration	[93]
		CDC25B	CDK1/cyclinB	Poor	RNA stability by YTHDF1	Proliferation	[191]
		CTSL		Poor	RNA stability by IGF2BP2	Metastasis	[94]
	Ovarian cancer	pri-miRNA-1246	CCNG2	Poor	promote pri-miRNA to mature	Proliferation, metastasis	[195]
		pri-miR-126-5p	PTEN, PI3K/Akt/mTOR		promote pri-miRNA to mature	Proliferation, migration, invasion	[194]
	Gallbladder cancer	DUSP5		Poor	RNA decay by YTHDF2	Proliferation, migration, invasion	[100]
	Esophageal squamous cell carcinoma	IFIT2		Poor		Proliferation, migration, invasion	[97]
	Renal cell carcinoma	ZNF667	CDKN3	Poor	RNA stability by IGF2BP2 RNA translation by YTHDF1	Proliferation	[197]
		HHLA2		Poor		Proliferation, migration, invasion	[96]
	Multiple myeloma	pri-miR-27a-3p, YY1	miR-27a-3p	Poor		Proliferation, stemness, apoptosis	[89]
	Osteosarcoma	LncRNA DANCR		Poor		Proliferation, migration, invasion	[90]
Tumor suppressor	Colorectal cancer	KIF26B			RNA decay by YTHDF2	Inhibit proliferation, migration	[151]
	Lung adenocarcinoma	FBXW7			RNA translation by ?	Tumor progression	[306]
	Thyroid carcinoma	STEAP2	Hedgehog			EMT	[105]

*Represents the correlation of a higher expression level of METTL13 with a good or poor prognosis

**Table 4:** The role of METTL14 in various cancers

Role	Cancer	METTL14 target	Downstream	Prognosis*	Mechanism	Cellular function	References
Oncogene	Pancreatic cancer	PERP		Poor	RNA decay by YTHDF2	Proliferation, migration	[208]
	Prostate cancer	THBS1		Poor	RNA decay by YTHDF2	Proliferation	[209]
	Hepatocellular carcinoma	HNF3*γ*	OATP1B1, OATP1B3	Poor	RNA stability by IGF2BPs	Sorafenib resistance	[210]
		Nedd4	TGF-*β*, TGFBR1			Growth, metastasis	[211]
		ARHGAP5-AS1	CSDE1, TRIM28		RNA stability by IGF2BP2	Invasion, metastasis	[307]
	Gastric cancer	LHPP	GSK3b, HIF1A			Growth, metastasis	[212]
	Breast cancer	E2F8			RNA stability by YTHDF1	Chemoresistance, DNA replication, DNA damage repair	[213]
	Head and neck squamous cell carcinoma	LNCAROD	YBX1, HSPA1A			Proliferation, mobility	[214]
	Bladder cancer	lncDBET	FABP5, PPAR		RNA decay by YTHDF2	Proliferation, migration	[229]
Tumor suppressor	Cholangiocarcinoma	MACF1			loss-of-function mutation R298H in METTL14	Proliferation, metastasis	[198]
		Siah2	PD-L1		RNA decay by YTHDF2	Immunotherapy sensitivity	[249]
	Colorectal cancer	SOX4	EMT, PI3K/AKT	Better	RNA decay by YTHDF2	Migration, invasion and metastasis	[124]
		XIST		Better	RNA decay by YTHDF2	Growth, invasion, Metastasis	[123]
		KLF4		Better	RNA stability by IGF2BP2	Metastasis	[199]
		ARRDC4	ZEB1	Better	RNA decay by YTHDF2	Metastasis	[155]
	Renal cell carcinoma	Pten	PI3K/AKT	Better	RNA stability by YTHDF1	Proliferation, migration	[200]
		ITGB4	EMT, PI3K/AKT	Better	RNA decay by YTHDF2	Migration, invasiveness, Metastasis	[201]
		BPTF	ENO2, SRC	Better		Metastasis	[230]
	Bladder cancer	USP38	EMT		RNA decay by YTHDF2	Migration, invasion	[202]
					Malfunction of the methyltransferase complex	Invasion, migration, apoptosis	[203]
	Gastric cancer	circORC5	miR-30c-2-3p/AKT1S1	Better		Growth, invasion	[125]
	Esophageal squamous cell carcinoma	miR‐99a‐5p	TRIB2, HDAC2, Akt/mTOR/S6K1			Radioresistance, cancer stem-like cells persistence	[205]
	Breast cancer	miR-375	SOX12			Proliferation, invasion	[161]
	Papillary thyroid cancer	OIP5-AS1	EGFR, AKT, MEK/ERK			Proliferation, migration, invasion	[207]

*Represents the correlation of a higher expression level of METTL14 with a good or poor prognosis

**Table 5:** Small-molecule inhibitors of the METTL3-14 in preclinical and clinical development

No.	Inhibitors	PubChem CID	MW (Da)	Clinical trials title	Phase	Status	ClinicalTrials.gov identifier
1	Quercetin	5,280,343	302.23	Effect of Quercetin in Prevention and Treatment of Oral Mucositis (Chemotherapy Induced Oral Mucositis)	Phase 2	Completed	NCT01732393
				Quercetin Chemoprevention for Squamous Cell Carcinoma in Patients With Fanconi Anemia	Phase 2	Recruiting	NCT03476330
				Reduce Senescence and Improve Frailty in Adult Survivors of Childhood Cancer	Phase 2		NCT04733534
				Therapeutic Efficacy of Quercetin Versus Its Encapsulated Nanoparticle on Tongue Squamous Cell Carcinoma Cell Line	Phase 2		NCT05456022
				Effect of Quercetin on Green Tea Polyphenol Uptake in Prostate Tissue From Patients With Prostate Cancer Undergoing Surgery	Phase 1	Completed	NCT01912820
				Trial of Quercetin in the Treatment and Prevention of Chemotherapy-induced Neuropathic Pain in Cancer Patients	Early Phase 1	Withdrawn	NCT02989129
				Sulindac and Plant Compounds in Preventing Colon Cancer	N/A	Terminated	NCT00003365
2	Delicaflavone	–					
3	STM2457	155,167,581	444.5	Create 2020-12-19			
4	UZH1a	154,815,692	558.7	Create 2020-11-24			
5	Eltrombopag	135,449,332	442.5	Identification and Validation of Biomarkers for Breast Cancer Resistance Protein (BCRP)	Phase 1	Recruiting	NCT04542382
				Study Impact on Outcome of Eltrombopag in Elderly Patients With Acute Myeloid Leukemia Receiving Induction Chemotherapy	Phase 2	Active, not recruiting	NCT03603795
				Pilot Trial of Eltrombopag in Patients Undergoing Chemotherapy for Malignant Solid Tumors	Phase 1	Recruiting	NCT04485416
				Eltrombopag Treatment in Patients With Prolonged BM Toxicity After CART	Phase 2	Recruiting	NCT05286164
				Phase II Eltrombopag in Chronic Lymphocytic Leukemia (CLL)	Phase 2	Completed	NCT01168921
				Eltrombopag for Chemotherapy-induced Thrombocytopenia	Phase 2	Recruiting	NCT04600960
				Eltrombopag in Elderly Acute Myelogenous Leukemia (AML)	Phase1/Phase2	Terminated	NCT01113502
				A Study of Eltrombopag in Patients With CMML and Thrombocytopenia	Phase1/Phase2	Completed	NCT02323178
				Dose Individualization of Antineoplastic Drugs and Anti-Infective Drug in Children With Hematoplastic Disease	Phase 4	Unknown	NCT03844360
				A Safety and Efficacy Study of Eltrombopag in Subjects With AML	Phase 2	Completed	NCT01890746
				Eltrombopag Used in Thrombocytopenia After Consolidation Therapy in AML	Phase2/Phase3	Unknown	NCT03701217
				Dose Finding Study Of Oral Eltrombopag In Patients With Sarcoma Receiving Adriamycin And Ifosfamide	Phase 1	Completed	NCT00358540
				A Three-part Study of Eltrombopag in Thrombocytopenic Subjects With Myelodysplastic Syndromes or Acute Myeloid Leukemia	Phase 2	Completed	NCT01440374
				A Randomized Placebo-controlled Phase 2 Study of Decitabine With or Without Eltrombopag in AML Patients	Phase 2	Unknown	NCT02446145
				Eltrombopag in Thrombocytopenic Chronic Lymphocytic Leukemia (CLL) Patients (CLL2S Study of GCLLSG)	Phase1/Phase2	Terminated	NCT01397149
6	CDIBA derivative	–					

Current research also indicates that multiple members of the methyltransferase-like protein family are abnormally highly expressed in cancer tissues, promoting tumor formation and progression. Therefore, a combined analysis of these protein expression levels can help assess patient prognosis. The highly expressed METTLs in tumor tissues have potential value as tumor biomarkers or therapeutic targets for designing effective small molecule inhibitors. These small molecule inhibitors have potential future clinical applications, and combining them with immunotherapy and chemotherapy drugs is significantly important for enhancing clinical efficacy.

## Data Availability

Not applicable.

## Abbreviations

m6A: *N*(6)-Methyladenosine
6mA: *N*(6)-Methyldeoxyadenine
SAM: *S*-Adenosyl methionine
METTL1: Methyltransferase-like 1
WDR4: WD repeat domain 4
m7G: *N*7-Methylguanosine
pri-miRNA: Primary miRNA transcript
METTL3: Methyltransferase-like 3
MTC: M6A methyltransferase complex
snRNA: Small nuclear RNA
pre-miRNA: Precursor miRNA
lncRNA: Long noncoding RNA
FCR1: FMR1 autosomal homolog 1
5mC: DNA 5-methylcytosine
TERRA: Telomeric repeat-containing RNA
ALT: Alternative lengthening of telomeres
PABPC1: Poly(A)-binding protein cytoplasmic 1
eIF4F: Eukaryotic translation initiation factor 4E
SCC: Spermatogonial stem cell
HSC: Hepatic stellate cells
TFAM: Transcription factor A
FBXW7: F-box and WD repeat domain
DDI2: DNA damage-inducible 1 homolog 2
eEF1AK55me2: EEF1A (eukaryotic elongation factor 1A) lysine 55
HN1L: Hematological and neurological expressed 1 like
HMGA2: High mobility group AT-hook 2
ARNT: Aryl hydrocarbon receptor nuclear translocator
TET1: Tet methylcytosine dioxygenase 1
RNAPII: RNA polymerase II
SOX4: SRY-related high-mobility-group box 4
EGR1: Early growth response 1
A-to-I: Adenosine-to-inosine
ADAR1: Adenosine deaminase RNA specific 1
ORF: Open reading frame
c-JUN: Jun proto-oncogene
PLAA: Phospholipase A2-activating protein
MRG3: Maternally expressed gene 3
AraC: Arabinocytosine
MRG3: Maternally expressed gene 3
SPI1: Spi-1 proto-oncogene
CDA: Cytidine deaminase
LPS: Lipopolysaccharide
NASH: Non-alcoholic steatohepatitis
HuR: Hu-Antigen R
TCF4: Transcription factor 4
AURKA: Aurora kinase A
EMT: Epithelial-mesenchymal transition
CLK1: Cdc2-like kinases 1
NBR1: Neighbor of BRCA1 gene 1
UVB: Ultraviolet B
piRNA: PIWI-interacting RNA
EBV: Epstein–Barr virus
EBNA3C: Epstein–Barr nuclear antigen 3C
KDM5C: Lysine specific histone demethylase 5C
PRMT1: Protein arginine methyltransferase 1
NHEJ: Non-homologous end joining
EGFR: Epidermal growth factor receptor
EFEMP1: Extra-cellular matrix protein 1
CCND3: Cyclin D3
RPTOR: Regulatory protein of MTOR complex 1
ULK1: Unc-51 like autophagy activating kinase 1
ZMYM1: Zinc finger MYM-type-containing 1
SPHK2: Sphingosine kinase 2
KLF2: KLF transcription factor 2
IGF2BP1: Insulin-like growth factor 2 mRNA-binding protein 1
CUL4B: Cullin 4B
KRT18: Keratin 18
DEK: DEK proto-oncogene
CRB2: Crumbs cell polarity complex component 2
EphA2: EPH receptor A2
VEGFA: Vascular endothelial growth factor A
SOCS2: Suppressor of cytokine signaling 2
YAP1: Yes1 associated transcriptional regulator
PARP: Poly (ADP-ribose) polymerase
YTHDF: YTH *N*6-methyladenosine RNA-binding protein
eIF3b: Eukaryotic translation initiation factor 3 subunit B
DAPK2: Death-associated protein kinase 2
H2AX: H2A histone family member X
ARHGDIA: Rho GDP dissociation inhibitor alpha
PTEN: Phosphatase and tensin homolog
ZNF217: Zinc finger protein 217
NANOGA: Nanog homeobox
STK4: Serine/threonine stress kinase 4
UBXN1: UBX domain protein 1
RBD: RNA-binding domain
CTNNBIP1: Beta-catenin-interacting protein 1
PTCH1: Protein patched homolog 1
GLI2: GLI family zinc finger 2
CDC25: Cyclin 25
CDK1: Cyclin-dependent kinases 1
CTSL: Cathepsin L
TXNDC5: Thioredoxin domain-containing protein 5
CCNG2: Cyclin G2
SLC7A11: Solute carrier 7A11
BIRC5: Baculoviral IAP repeat-containing 5
DUSP5: Dual-specificity phosphatase 5
IFIT2: Interferon-induced protein with tetratricopeptide repeats 2
ZNF667: Zinc finger protein 677
HHLA2: HERV-H LTR-associating 2
YY1: Yin Yang 1
HDAC5: Histone deacetylase 5
ARMC8: Armadillo1340 repeat-containing 8
MACF1: Microtubule actin crosslinking factor 1
SOX4: SRY1341-related high-mobility-group box 4
KLF4: Kruppel-like factor 4
ITGB4: Integrin 4
USP38: Ubiquitin-specific peptidase 38
TRIB2: Inhibited tribble 2
THBS1: Thrombospondin 1
HNF3*γ*: Hepatocyte nuclear factor 3*γ*
Nedd4: NEDD4 E3 ubiquitin protein ligase
TGFBR1: TGF-*β* type I receptor
ACLY: ATP citrate lyase
SCD1: Stearoyl-CoA desaturase
SLP2: Stomatin-like protein 2
NUDFA4: NADH dehydrogenase (ubiquinone) 1 alpha subcomplex 4
HIF-1*α*: Hypoxia-inducible factor 1 subunit alpha
LDHA: Lactate dehydrogenase A
TGF-*β*1: Transforming growth factor *β*1
DM2: Type 2 diabetes mellitus
SCD1: Stearoyl-CoA desaturase1
BPTF: Bromodomain PHD finger transcription factor
ENO2: Enolase 2
SRC: SRC proto-oncogene
USP48: Ubiquitin-specific peptidase 48
PMN-MDSCs: Polymorphonuclear-myeloid derived suppressor cells
CXCL8: Interleukin-8
LXA4: Lipoxin A4
Jak1: Janus kinase 1
BHLHE41: Basic helix-loop-helix family member e41
CXCL1: C-X-C motif chemokine ligand 1
Ebi3: Epstein–Barr virus-induced protein 3
TICAM2: TIR domain-containing adaptor molecule 2
TLR4: Toll-like receptor 4
LATS2: Large tumor suppressor kinase 2
APAF1: Apoptotic protease activating factor 1
RAD51AP1: RAD51-associated protein 1
IFFO1: Intermediate filament family orphan 1
CPEB2: Cytoplasmic polyadenylation element-binding protein 2
SRSF5: Serine and arginine rich splicing factor 5
GPX4: Glutathione peroxidase 4
HMOX1: Heme oxygenase 1
SOD1: Superoxide dismutase 1
ATRA: All-trans-retinoic acid
MN1: Meningioma 1
E2F8: E2F transcription factor 8
PHLDB2: Pleckstrin homology-like domain, family B, and member 2
TPO-R: Thrombopoietin receptor
m4C: *N*4-Methylcytidine
ETF*β*: *β* Subunit electron transfer flavoprotein
1MH: 1-Methylhistidine
RPL3: Ribosomal protein L3
